# Dysregulation of AGO2-miRNA dynamics underlies the *AGO2*-associated Lessel–Kreienkamp syndrome

**DOI:** 10.1093/nar/gkaf1002

**Published:** 2025-10-16

**Authors:** TingYu M Liu, Debora Tibbe, Jan Broder Engler, Irm Hermans-Borgmeyer, Uwe Borgmeyer, Kerstin Robles de Maruri, Davor Lessel, Ian J MacRae, Hans-Jürgen Kreienkamp

**Affiliations:** Department of Integrative Structural and Computational Biology, The Scripps Research Institute, La Jolla, CA 92037, United States; Institute of Human Genetics, University Medical Center Hamburg-Eppendorf, Hamburg 20246, Germany; Institute for Neuroimmunology and Multiple Sclerosis, at the Center for Molecular Neurobiology, University Medical Center, Hamburg-Eppendorf 20251, Germany; Center for Molecular Neurobiology, University Medical Center Hamburg-Eppendorf, Hamburg 20251, Germany; Center for Molecular Neurobiology, University Medical Center Hamburg-Eppendorf, Hamburg 20251, Germany; Institute of Human Genetics, University of Regensburg, Regensburg 93053, Germany; Institute of Human Genetics, University Medical Center Hamburg-Eppendorf, Hamburg 20246, Germany; Institute of Human Genetics, University of Regensburg, Regensburg 93053, Germany; Institute of Clinical Human Genetics, University Hospital Regensburg, Regensburg 93053, Germany; Department of Integrative Structural and Computational Biology, The Scripps Research Institute, La Jolla, CA 92037, United States; Institute of Human Genetics, University Medical Center Hamburg-Eppendorf, Hamburg 20246, Germany

## Abstract

Mutations in human Argonaute genes, *AGO1* and *AGO2*, are associated with neurodevelopmental disorders. Although multiple patients have been identified, the underlying molecular basis for pathogenesis remains unclear. Here, we biochemically examined five *AGO2* mutations (p.L192P, p.A367P, p.T357M, p.F182del, and p.G733R) linked to different clinical severities. Except for G733R, all AGO2 mutant proteins maintained a stable fold, capable of binding and using microRNA (miRNA) guides. Kinetic studies showed that the L192P, A367P, and T357M mutants have prolonged dwell times on target RNAs, indicating impaired target release. The L192P and A367P variants also display slow target RNA association kinetics. RNA Bind-n-Seq experiments showed that *in vitro*, the L192P, A367P, T357M, and F182Δ mutants are prone to mis-targeting. In cultured murine cortical neurons, the L192P mutant altered the miRNA complement associated with AGO2, altered guide strand selectivity, and increased the accumulation of 3′ isomiRs, suggesting altered miRNA loading and increased miRNA 3′ end exposure. *In vivo*, mice carrying the p.L192P variant, but not p.G733R, demonstrated strongly reduced breeding ability, altered cortical transcriptomes, and over-repression of miRNA targets. The combined results suggest patient mutations impact AGO2 targeting dynamics in a gain-of-function manner, leading to deregulation of the neuronal transcriptome and the observed neurodevelopmental anomalies.

## Graphical Abstract

**Graphical Abstract:**
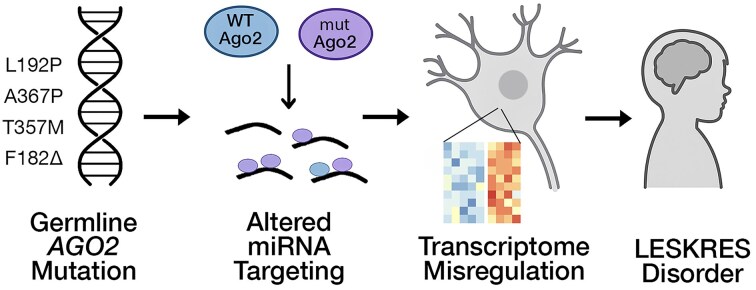

## Introduction

Next-generation sequencing (NGS) technologies are now routinely used to identify potentially causative variants for a broad variety of Mendelian disorders, including neurodevelopmental disorders (NDDs). However, despite these advancements, two significant challenges persist for most NDDs: accurately determining the pathogenicity of newly identified variants and translating genetic findings into effective clinical interventions. Addressing both challenges requires a thorough understanding of the molecular and cellular consequences of the identified mutations.

Recently, heterozygous *de novo* mutations in the human Argonaute genes *AGO1* and *AGO2* have been associated with similar, ultra-rare NDDs [1, 2], namely *Neurodevelopmental disorder with language delay and behavioral abnormalities, with or without seizures* (NEDLBAS; #620 292) and *Lessel-Kreienkamp syndrome* (LESKRES; # 619 149), respectively. Argonaute proteins (AGO1-4) are essential mediators of RNA interference, assembling with miRNAs to form the RNA-induced silencing complex (RISC) [3]. Additionally, siRNAs can also be incorporated into Argonaute proteins via RISC assembly. RISC assembly thereby serves as the central point of convergence between the miRNA and siRNA pathways. However, in mammals, the siRNA pathway plays an unclear role in terms of physiology. Once a small guide miRNA duplex is loaded into an Argonaute protein, one of the two strands remains and is termed the guide, whereas the other strand, termed the passenger, is released and degraded. The Argonaute protein in complex with the guide binds to cognate sites in target messenger RNAs (mRNAs). The outcome of binding depends on the degree of complementarity between the guide RNA and the target mRNA. Targets with perfect or near-perfect complementarity can be directly cleaved by AGO2 and AGO3 [4–6]. However, for miRNAs, it is more common to recognize partially complementary target sites, which leads to translational repression and accelerated mRNA decay [7]. Argonaute proteins accomplish these effects by recruiting GW182, which interacts with PABP and deadenylation and decapping enzymes, ultimately contributing to mRNA degradation [8]. Given its critical role in gene expression regulation, the miRNA pathway has significant implications for the nervous system. Dysregulation at various stages of miRNA metabolism can impact neuronal morphology, synapse formation, synaptic plasticity, and key processes related to learning and memory [9–11].

Previous functional characterization of *AGO2* mutations associated with LESKRES revealed a shared defect in short hairpin RNA (shRNA)-mediated silencing, suggesting that direct target cleavage is impaired [2]. Additionally, mutation-specific molecular effects were observed, wherein the missense mutation p.G733R impaired RISC formation. Other *AGO2* mutations did not affect fundamental aspects of RISC but exhibited increased binding to mRNA targets, reduced phosphorylation of a *C*-terminal serine cluster involved in mRNA target release, and enhanced formation of dendritic P-bodies in neurons [2].

Here, to further elucidate the molecular mechanisms underlying LESKRES, we conducted an *in-depth* functional characterization of selected *AGO2* mutations. We analyzed the protein stability, RNA binding affinity, and dwell times on target RNAs, along with target binding and mis-targeting rates. The mutation resulting in the most severe deficits, p.L192P, was further investigated using cortical murine neurons and knock-in mice, employing both morphological analyses and NGS-based techniques.

## Materials & methods

### Cloning and mutagenesis for generating Ago2 mutant proteins

Plasmids encoding AGO2 mutants were produced using NEBuilder® HiFi DNA Assembly. PCR was used to generate 5′ and 3′ fragments of mutant AGO2 constructs using the primers listed in Supplementary Table S2 with a wild-type cDNA clone of AGO2 as a template. PCR products were purified by agarose gel electrophoresis and assembled with a modified version of pFastBac (HTA) that had been linearized by digestion with *Sfo*I and *Xho*I. All constructs also carried mutations at AGO2 phosphorylation sites (S387D, S824A, S828D, S831D, and S834A) to avoid indirect effects associated with differential phosphorylation during protein expression.

### Bacterial strains and plasmids for mutant AGO2 protein production

Chemically competent *Escherichia coli* OmniMAX^T^ (C854003, Thermo Fisher) were used for all molecular cloning steps. Bacmid DNAs were produced using DH10Bac *E. coli* (10 361 012, Thermo Fisher).

### Bacterial media and growth conditions for mutant AGO2 protein production

Bacterial cultures were typically grown in Luria–Bertani (LB) medium at 37°C. For plasmid selection, media was supplemented with one or more of the following: ampicillin (100 μg/mL), kanamycin (40 μg/mL), tetracycline (5 μg/mL), gentamycin (7 μg/mL), 5-Bromo-4-Chloro-3-Indolyl β-D-Galactopyranoside (X-gal, 25 μg/mL in dimethylformamide), and/or Isopropyl β-D-1-thiogalactopyranoside (IPTG, 1 mM).

### Insect cell media and growth conditions for mutant AGO2 protein production

Sf9 cells (94–001S, Expression Systems) were grown in Lonza Insect XPRESS medium supplemented with 1x Gibco Antibiotic-Antimycotic at 27°C in monolayer dishes, or in suspension with moderate shaking.

### AGO2-guide RNA complexes preparation

All AGO2 proteins were expressed in Sf9 cells using a baculovirus system (Invitrogen) as described previously [12]. For each protein, 750 mL of Sf9 cells at 2.0 × 10^6^ cells/mL were infected with 7.5 mL baculovirus and incubated at 27°C for ∼60 h with aeration via shaking. Infected cells were then harvested by centrifugation. Each cell pellet was suspended in ∼15 mL Lysis Buffer (50 mM Tris pH 8.0, 300 mM NaCl, 0.5 mM TCEP). Resuspended cells were lysed by passing five times through an M-110P lab homogenizer (Microfluidics). The resulting total cell lysate was clarified by centrifugation (15 000 rpm for 20 min at 4°C), and the supernatant fraction of each sample was applied to 3 mL packed Ni-NTA resin (Qiagen) and gently rocked at 4°C for 1 h in 50 mL conical tubes. The resin was pelleted by brief centrifugation and the supernatant solution was discarded. The resin was washed with ∼45 mL ice-cold Nickel Wash Buffer (50 mM Tris pH 8.0, 300 mM NaCl, 20 mM Imidazole, 0.5 mM TCEP). Centrifugation/wash steps were repeated once more with Nickel Wash Buffer, followed by an additional wash supplemented with 5 mM CaCl_2_. Around 100U of micrococcal nuclease (Clontech) was added to the washed resin, and the resulting mixture was incubated at room temperature for 1 h to reduce the amount of co-purifying nucleic acids in the preparation. The nuclease-treated resin was washed twice again with Nickel Wash Buffer and then eluted in three fractions (7.5, 5, and 5 mL) using Nickel Elution Buffer (50 mM Tris pH 8.0, 300 mM NaCl, 300 mM Imidazole, 0.5 mM TCEP). EGTA was added to the eluted protein to a final concentration of 5 mM. Eluted protein was then supplemented with 5 nmol synthetic guide RNA (miR-122, Supplementary Table S2) and 150 μg TEV protease. The resulting mixture was dialyzed against 1 liter of Dialysis Buffer (50 mM Tris pH 8.0, 300 mM NaCl, 15mM Imidazole, 0.5 mM TCEP) at 4°C overnight.

The following day, AGO2–miR-122 complexes were purified away from AGO2 molecules associated with small RNAs endogenous to sf9 cells using a modified version of the Arpon method [13]. First, AGO2-miR-122 capture resin was prepared by incubating 144 μL packed High Capacity Neutravidin Resin (Thermo Fisher) with 12 nmol biotinylated miR-122 capture oligo in Wash A Buffer (30 mM Tris pH 8.0, 100 mM KOAc, 2 mM MgOAc, 0.02% CHAPS, 0.5mM TCEP) for 30 min at 4°C. Unbound capture oligo was removed by washing the resin with 50 mL Wash A Buffer. Dialyzed Ago2 protein was recovered from dialysis and supplemented with 0.02% CHAPS (final concentration) and 2 mM MgOAc (final concentration). The resulting mixture was then incubated with capture resin at RT for 2 h. Unbound material was removed and the resin was washed twice with 10 mL Wash A followed by five times with 10 ml Wash B Buffer (30 mM Tris pH 8.0, 2 M KOAc, 2 mM MgOAc, 0.02% CHAPS, 0.5mM TCEP) and twice with Wash C Buffer (30 mM Tris pH 8.0, 1 M KOAc, 2 mM MgOAc, 0.02% CHAPS, 0.5mM TCEP). The AGO2-guide sample was then eluted by resuspending the resin in 720 μL Wash C Buffer containing 24 nmol of the competitor DNA and incubating at RT for 30 min with gentle shaking. The eluted sample was then incubated with 144 μl (packed) of fresh High Capacity Neutravidin Resin (equilibrated in 50mM Tris pH 8.0, 150mM NaCl, 0.5mM TCEP) at RT for 30 min to remove excess competitor DNA. The supernatant solution (containing the AGO2–miR-122 complexes) was then buffer exchanged into Q Dialysis Buffer (30 mM Tris pH 8.0, 150 mM NaCl, 0.5 mM TCEP) using a 4 ml concentrator (30 kDa MWCO). During buffer exchange, 80 μl of Q Sepharose Fast Flow anion exchange resin slurry (GE Healthcare) was equilibrated in Q dialyzing buffer. The buffer-exchanged AGO2-miR-122 sample was then incubated with this resin for 30 min to remove remaining unbound oligonucleotides. The flow-through solution was collected and concentrated to ∼1 mg/mL in Q Dialysis Buffer. The concentrated protein was aliquoted and stored at −80°C. The concentration of the AGO2–guide RNA complex was determined by Nanodrop using the extinction coefficient ϵ_280_ = 198 370 M^−1^ cm^−1^ (assuming a molecular weight of 100 kDa).

### Equilibrium target binding assays

Equilibrium target binding assays were performed in a similar manner described previously [14]. Various concentrations (0–10 nM) of wild-type and mutant AGO2-miR-122 samples were incubated with ∼0.1 nM 5′ ^32^P‐radiolabeled target RNA in binding reaction buffer (30 mM Tris pH 8.0, 100 mM KOAc, 2 mM MgOAc, 0.5 mM TCEP, 0.005% (v/v) NP‐40, 0.01 mg/ml baker’s yeast tRNA) with a total volume of 50 μl for 2 h at RT. The reactions were then applied to a vacuum-connected dot-blot apparatus (GE Healthcare Life Sciences), with Protran nitrocellulose membrane (0.45‐μm pore size, Whatman, GE Healthcare Life Sciences) capturing the protein–RNA complexes, and Hybond Nylon membrane (Amersham, GE Healthcare Life Sciences) capturing the unbound target RNA. Each applied dot was immediately washed with 50 μl wash buffer (30 mM Tris pH 8.0, 100 mM KOAc, 2 mM MgOAc, 0.5 mM TCEP). Membranes were air-dried and radioactive signals were visualized by phosphorimaging. ImageQuant (GE Healthcare Life Sciences) was used to quantify signal data and dissociation constants were calculated using Prism version 6.0g (GraphPad Software, Inc.), using the following formula, which accounts for potential ligand depletion [15]:


\begin{eqnarray*}
{\mathrm{F = Bmax}}\frac{{\left( {\left[ {{\mathrm{ET}}} \right]{\mathrm{ + }}\left[ {{\mathrm{ST}}} \right]{\mathrm{ + KD}}} \right){\mathrm{ - }}\sqrt {{{{\left( {\left[ {{\mathrm{ET}}} \right]{\mathrm{ + }}\left[ {{\mathrm{ST}}} \right]{\mathrm{ + KD}}} \right)}}^{\mathrm{2}}}{\mathrm{ - 4[ET][ST]}}} }}{{{\mathrm{2[ST]}}}}
\end{eqnarray*}

where F is the fraction of target bound, B_max_ is the calculated maximum number of binding sites, [E_T_] is the total enzyme concentration, [S_T_] is the total target concentration, and *K_D_* is the apparent equilibrium dissociation constant.

### Target dissociation assays

Target dissociation rates were determined by mixing 2.5 nM of a AGO2-miR-122 sample with ∼0.1 nM ^32^P 5′-radiolabeled target RNA in binding buffer (30 mM Tris pH 8.0, 100 mM potassium acetate, 2 mM Magnesium acetate, 0.5 mM TCEP, 0.005% (v/v) NP-40, 0.01 mg/mL baker’s yeast tRNA) in a single reaction mixture with a volume of 50 μL per time point planned for the experiment (for example, 300 μl for six time points). The resulting mixture was incubated at room temperature for 60 minutes to allow binding to approach equilibrium. After equilibration, a zero-time point was taken by applying 50 μl of the reaction to the dot-blot apparatus fit with a Protran nitrocellulose membrane on top of a Hybond Nylon membrane under vacuum. The applied sample was immediately washed with 50 μL of ice-cold wash buffer (30 mM Tris pH 8.0, 100 mM potassium acetate, 2 mM Magnesium acetate, 0.5 mM TCEP). The dissociation time course was started by the addition of 300 nM (final concentration) unlabeled target RNA. Aliquots of 50 μL were taken at various times and immediately applied to the dot-blot apparatus under vacuum, followed by 50 μL of ice-cold wash buffer. Time points ranged from 1 to 16 min. Membranes were air-dried and visualized by phosphorimaging. ^32^P signal was quantified using ImageQuant TL (GE Healthcare). The fraction of target RNA bound was calculated as the ratio of bound to total (bound + free) target RNA. Dissociation rates were calculated by plotting data as fraction bound versus time and fitting to a two-phase decay curve using Prism v.8.0 (GraphPad).

### Target association assay

Target association experiments were set up by first incubating ∼5 pM ^32^P 5′-radiolabeled target RNA (Supplementary Table S2) in binding reaction buffer (30 mM Tris pH 8.0, 100 mM potassium acetate, 0.5 mM TCEP, 0.005% (v/v) NP-40, 0.01 mg ml^−1^ baker’s yeast tRNA) in a single large reaction with a volume of 50 μl for each time point planned for the experiment at room temperature for 15 min. A zero-time point was taken by applying 50 μl of the reaction to the dot-blot apparatus fit with a Protran nitrocellulose membrane on top of a Hybond Nylon membrane under vacuum. The applied sample was immediately washed with 50 μL of ice-cold wash buffer (30 mM Tris pH 8.0, 100 mM potassium acetate, 2 mM Magnesium acetate, 0.5 mM TCEP). The association reaction was then initiated by the addition of AGO2-miR-122 at concentrations ranging from 0.25 to 1.5 nM. 50 μl aliquots were taken at various times and applied to a dot-blot apparatus under vacuum, followed by 50 μl of ice-cold wash buffer. Time points ranged from 15 to 300 s. Membranes were air-dried and visualized, the signal was quantified, and the bound fraction was calculated as described above for target dissociation assays. Data were fit to a one-phase exponential curve using Prism v.8.0 (GraphPad) to determine an observed binding rate constant (*k_on,_*_obs_). *k_on,_*_obs_ were then replot as a function of AGO2-miR-122 concentration and the slopes of the resulting lines were taken as the association rate constants (*k*_on_).

### AGO2 bind-n-seq experiment

A pool of 256 target RNAs that contain the sequence “RYRYYYYR,” where R is purine and Y is pyrimidine, flanked by constant sequences was synthesized by Integrated DNA Technologies (Supplementary Table S2). This pattern of purines and pyrimidines contains the sequence “ACACTCCA,” which is a perfect match (8mer) to the miR-122 seed sequence, as well as 255 mismatched variations of the 8mer. The target RNA pool was first annealed to the 5′ and 3′ blocker oligos, which are complementary to the constant regions of the target RNAs, minimizing secondary structure. Annealing was accomplished by mixing the target RNA library (1μM) with blocker oligos (1.2 μM each) in a buffer of 120 mM potassium acetate, 3.5 mM magnesium acetate, and 30 mM Tris, pH 8 and incubating the resulting mixture at 90°C for 5 min. The heated sample was then placed on the benchtop and allowed to cool back to room temperature. The target RNA library was diluted to a concentration of 100 nM using Binding Reaction Buffer (30 mM Tris pH 8.0, 0.1 M potassium acetate, 2 mM magnesium acetate, 0.5 mM TCEP, 0.005% NP-40, and 0.01 mg/ml yeast tRNA).

The binding reaction was initiated by the addition of AGO2–miR-122 complex (3 nM, final concentration). The final reaction volumes were 100 μl. After 30 s, half of each binding reaction (50 μl) was applied to a Protran nitrocellulose membrane in a Dot Blot apparatus under vacuum to capture AGO2-bound RNAs. The spot was immediately washed with 50 μl of Binding Reaching Buffer (minus tRNA) to ensure only AGO2-associated RNA remained on the membrane. The second half of each binding reaction was treated the same way after incubating for 60 min. Spots of the membrane containing RNA were excised with a razor blade and target RNAs were recovered by incubating each cutout spot in a 1.5 ml tube containing 300 μl of 100mM Tris, 200mM NaCl, 5mM EDTA, 1% SDS, and incubating at 45°C for an hour. Each extracted RNA solution was then mixed with 300 μl of phenol:chloroform:isoamyl alcohol (25:24:1 ratio). After centrifugation, the top layer was moved to a new tube and mixed with 30 μl of 3 M sodium acetate, pH 5.2, and 750 μl of ice-cold ethanol. The resulting mixture was incubated at −80 °C for at least 2 h. RNAs were precipitated by centrifugation, pellets washed with 70% ethanol, air-dried, and then resuspended in 20 μl of water.

The RNAs (and input library) were then transcribed into DNA using Superscript III reverse transcriptase (ThermoFisher) following the manufacturer's protocol and amplified by PCR using KAPA HiFi HotStart ReadyMix (Roche) and barcoded primers that add Illumina adapter sequences. PCR samples were resolved by 10% polyacrylamide gel electrophoresis in TBE buffer, and the desired products were excised with a razor blade. DNA was extracted from the gel slices, purified using Oligo Clean and Concentrator columns (Zymo Research), and analyzed by Illumina Sequencing. After sequencing, unique reads were trimmed of the adapter sequences and those of the correct read length were retained. The remaining reads were sorted into subsets corresponding to each experimental sample using the eight-base barcodes. A read count analysis was performed on each subset, and the fraction of the total number of reads for each sequence in each subset was determined. The fold enrichment was calculated by dividing the read fraction of each sequence in every sample by the read fraction of the corresponding sequence in the input library.

### Primary cell culture of mouse cortical neurons

A pregnant mouse was anaesthetized with CO_2_ and sacrificed by cervical dislocation. The embryos (E18) were isolated and cortices prepated in HBSS pen/strep. The tissue was treated with papain neuron isolation enzyme (Thermo Fisher Scientific, Waltham, USA) for 30 min and 37°C. The tissue was washed five times and triturated. The cells were counted and seeded on PLL-coated 6-well dishes at a density of 1 million cells per well. After 24 h of incubation at 37°C, in 5% CO_2_ and humidified air, the neuron plating medium was replaced by neuron culture medium and the neurons were cultured for 12–14 days.

### Transduction of cortical neurons with adeno-associated viruses

PAAV-hSyn1-EGFP-CW3SL, pAAV-hSyn1-EGFP-AGO2-WT-CW3SL, and pAAV-hSyn1-EGFP-AGO2-L192P-CW3SL plasmids were generated to induce expression of GFP empty vector control, GFP-tagged AGO2 wild-type (WT), and GFP-tagged AGO2-L192P. Adeno-associated viruses (AAVs) of the serotype PHP-eB were produced by the Vector Facility of the UKE Hamburg using the appropriate packaging plasmid (plasmid no. 103 005; Addgene, Watertown, USA) [16]. The AAVs were applied in of 25 000 MOI. 24 million neurons were transduced. Twenty-four hours after plating the murine cortical neurons, the neuronal plating medium was replaced with AAV-containing neuron culture medium. AAV-infected neurons were cultivated for another 11 days *in vitro* (DIV12).

### SDS page and western blot

Separation gels of 10% were poured with stacking gels of 1 cm width. 20 μl of samples were added to the wells. Around 80 V was applied for 10 min and then the voltage was increased to 160 V. Proteins were transferred to a nitrocellulose membrane using the wet blot method with 100 V applied for 2 h. For blocking, the nitrocellulose membranes were incubated in 10 ml of 5% MP in TBS-T for 30 min at RT. This was followed by incubation with the primary antibody in 5% MP in TBS-T at 4°C with rotation overnight. Membranes were washed three times with TBS-T for 10 min at RT and incubated with horseradish peroxidase coupled secondary antibody in TBS-T for 1 h at RT. Again, the membranes were washed three times with TBS-T and then incubated in chemiluminescent substrate (WesternBright from Biozym, Hamburg, Germany) before detection of the protein bands with an imaging system (BioRad, Munich, Germany).

### Immunofluorescent labelling and confocal microscopy

Neurons were transduced at 1 DIV and fixed at 12 DIV. Fixation was performed with PFA. Cells were washed three times in phosphate-buffered saline (PBS) and permeabilised with 0.1% (v/v) Triton X-100 in PBS for 3 min at RT. After washing three times with PBS, cells were incubated for 1 h with 10% HS in PBS. Primary antibodies were prepared in 2% HS in PBS. Around 100 μl drops of primary antibody solution were pipetted onto parafilm and coverslips were incubated at 4°C overnight. After washing three times with PBS for 10 min at RT, the coverslips were incubated with the secondary antibodies in PBS for 1 h at RT. Following three washes with PBS, the coverslips were washed once with ddH2O and mounted on slides using ProLong Diamond Antifade mounting medium (Thermo Fisher Scientific, Waltham, USA). Samples were stored at RT overnight to dry and analyzed using a SP8 confocal microscope with a 63x oil immersion objective (Leica, Wetzlar, Germany), provided by the UKE Microscopy Imaging Facility (UMIF, Hamburg).

### MicroRNA affinity purification

Preparation of beads: The microRNA Tagging and Affinity-purification (miRAP) was adapted from He 2012 [17]. 300 μl of Streptavidin MyOne T1 Dynabeads (Thermo Fisher Scientific, Waltham, USA) were resuspended in 900 μl of PBS. Around 120 μl of Biotinylated Pierce Recombinant Protein L (Thermo Fisher Scientific, Waltham, USA) was added, and the mixture was rotated for 35 min at RT. After coating, beads were washed five times with 3% IgG + Protease-free bovine serum albumin (BSA; Jackson ImmunoResearch, Ely, UK) in PBS. Next, beads were resuspended in 930 of μl 0.15 M KCl IP wash buffer. Monoclonal antibodies HtZ-GFP 19C8 and HtZ-GFP 19F7 (Heintz Lab, Rockefeller University, Cat# Htz-GFP-19F7, RRID:AB_2716736) were thawed on ice and centrifuged at 13 000 g and 4°C for 10 min. Around 50 μg of each anti-GFP antibody was added to the beads and rotated slowly at RT for 1 h. Beads were washed three times in 900 μl of 0.15 M KCl IP wash buffer and then resuspended in 200 μl of 0.15 M KCl IP wash buffer. Preparation of neurons: Cortical neurons from mice were infected with AAVs at 1 DIV. The neurons were washed three times in PBS and scraped from the culture dishes in 500 μl of RNase-free PBS per well. After centrifugation at 2000 g and 4°C for 5 min, the obtained cell pellet was resuspended in 1 ml of lysis buffer and incubated for 15 min on ice. Cell lysates of 22 million neurons were combined to generate one sample, divided into IC and IP. Neuron lysates were centrifuged at 2000 g and 4°C for 10 min and the supernatant transferred. 100 μl of 10% (v/v) NP-40 in ddH_2_O was added and mixed by inversion. Then, 100 μl of 300 mM 1,2-diheptanoyl-sn-glycero-3-phosphocholine (DHPC; Sigma-Aldrich, Taufkirchen, Germany) was added and again mixed by inversion. Samples were incubated on ice for 5 min and centrifuged for 10 min at 4°C and 20 000 g. The supernatant was again transferred. Around 100 μl was taken as IC. For immunoprecipitation, 200 μl of prepared beads was added to the sample and incubated overnight at 4°C with gentle rotation. The beads were washed four times with 0.35 M KCl wash buffer and resuspended in 700 μl of QIAzol (Qiagen, Hilden, Germany). Simultaneously, 700 μl of QIAzol was added to the IC samples and from then on IC and IP samples were processed in parallel. After incubation at RT for 5 min, 140 μl of chloroform was added and the samples were shaken vigorously for 15 s. An incubation at RT for 3 min as well as a centrifugation step at 12 000 g for 15 min at 4°C followed, which led to phase separation of a colorless aqueous RNA phase, a white interphase containing DNA, and an organic phase below that contained proteins and lipids. The upper phase was extracted to which 525 μl ≥ 99.8% of EtOH was added and mixed. For subsequent RNA purification, the miRNeasy Micro Kit (Qiagen, Hilden, Germany) was used according to the manufacturer’s protocol. The miRNA concentration was determined with the Qubit microRNA Assay-Kit (Thermo Fisher Scientific, Waltham, USA) and quantitative PCR (qPCR) on miRNAs was applied using the TaqMan Advanced miRNA Assay (Thermo Fisher Scientific, Waltham, USA) following manufacturer’s instructions. The relative enrichment (ΔCt) of miRNAs was calculated as Ct of the IP samples normalised to the Ct value measured in IC with ΔCt = [Ct(IP) - Ct(IN)].

### Generation and genotyping of AgoL193P and AgoG734R knock-in mice

For the generation of mice expressing *Argonaute* with the p.L193P knockin, a single-guide RNA (sgRNA) was chosen after submitting the targeting region around exon 5 to the CRISPOR design tool (http://crispor.tefor.net/) [18]. The template for transcription with the targeting sequence (TTT CCA AAG AGA AGG TCG GA) was generated by fill-in reaction with Klenow DNA Polymerase (Thermo Fisher Scientific). Transcription was performed using the HiScribe™ T7 High Yield RNA Synthesis Kit (#E2040S, New England Biolabs), with subsequent purification of the transcript with the MEGAClear™ Transcription Clean-Up Kit (#AM1908, Thermo Fisher Scientific), both according to the manufacturer’s instructions. A 120 bp Homology-Directed Repair (HDR) template (Sigma-Aldrich) designed to knock-in the p.L193P substitution and additional silent mutation of the PAM sequence to suppress Cas9 cleavage activity (Supplementary Table S1).

This donor DNA (1 μg/μL), sgRNA (600 ng/μL), and Cas9 protein (Alt-R® S.p. Cas9 Nuclease V3, #1 081 058, Integrated DNA Technologies (IDT), Leuven, Belgium) (500 ng/μL) in Gibco™ Opti-MEM™ (Thermo Fisher Scientific) were used for electroporation into one-cell-stage embryos derived from superovulated C57BL/6JUke mice using the NEPA 21 electroporator (Nepa Gene, Ichikawa-City, Japan; for settings, see [19]) and implanted into foster mice.

The *Ago^L193P^* knock-in mice were genotyped by PCR using genomic tail DNA. Primers were designed to amplify a 354-bp fragment containing the mutated sites (Supplementary Table S2). PCR products were enzymatically cleaned up using ExoSAP-IT™ (Applied Biosystems) for 15 min at 37°C, following 15 min at 85°C. Afterwards, the purified samples were analyzed by Sanger sequencing.

*Ago* ^G734R^ knock-in mice were generated in a similar manner, using the sequence AAG AGT GGG AAC ATT CCC GC for targeting. Again a 120-bp repair template was used, carrying the desired variant. For genotyping, a 550-bp segment of genomic DNA was amplified and sequenced (see Supplementary Table S2 for primer sequences).

### Small RNA and mRNA sequencing

Small RNA sequencing was performed by the NGS Integrative Genomics Core Unit (NIG) in Göttingen. Small RNA sequencing libraries were prepared using the TruSeq Small RNA Library Prep Kit (Illumina). Libraries were sequenced on an Illumina HiSeq 4000 machine generating 50 base pair single-end reads. mRNA sequencing libraries were prepared using Illumina Stranded mRNA Prep Kit (Illumina), and sequenced with a NextSeq 500/550 High Output Kit (illumina) on an Illumina NextSe550. by Eurofins Genomics, Ebersberg, Germany or at the Institute of Human Genetics, University of Regensburg, Regensburg, Germany.

*Small RNA-seq analysis*. The raw sequencing reads were adaptor-trimmed, aligned and counted with *OASIS 2.0* [20] (https://oasis.ims.bio/) using default parameters. miRNA counts were analyzed by *DESeq2* [21] (v1.36.0) calling miRNAs with an FDR-adjusted *P* < 0.05 and a log2 fold change > 1 differentially expressed. Expression heatmaps were generated from expression values after variance stabilizing transformation (*DESeq2*) using the R package *tidyheatmaps* (v0.2.1).

*mRNA-seq analysis*. The raw sequencing reads were aligned to the Ensemble mouse reference genome (GRCm39) using STAR (v2.7.9b) with default parameters and overlap with annotated gene loci were counted with *featureCounts* (v1.5.1). Samples were analyzed with *DESeq2* (v1.36.0) considering mRNAs with an FDR-adjusted *P* < 0.05 differentially expressed.

*miRNA–mRNA network analysis*. Predicted targets of the 9 up- and 14 downregulated miRNA candidates were extracted from *TargetScanMouse 8.0*. The confidence level of predictions was stratified by using the cumulative weighted context score (CWCS). Moderate (CWCS between –0.2 and –0.4) and high confidence targets (CWCS lower than –0.4) were retained and further filtered to only include mRNAs that were significantly regulated in the mRNA-seq analysis. Predicted interactions from this approach were used to construct a miRNA–mRNA network using the R packages *network* (v1.18.2) and *ggnetwork* (v0.5.13).

### Gene set enrichment analysis

To integrate Ago2 targets with mRNA expression changes in Ago2-L192P, we compiled a gene set containing 661 mRNAs derived from UTR SAP-seq peaks of P90 cortex samples (GEO: GSE289736). We generated an mRNA expression signature of Ago2-L192P versus WT controls generated by ranking all expressed genes by the DESeq2-derived *t* statistics. Then, we tested the enrichment of SAP-seq-derived Ago2 targets against the mRNA expression signature by gene set enrichment analysis (GSEA) using the R package *clusterProfiler* (v4.12.6).

### Animal experiments

Animal experiments were approved by Behörde für Justiz und Verbraucherschutz, Freie und Hansestadt Hamburg, Germany.

## Results

### The majority of LESKRES mutants are biochemically stable

Previous results suggest that LESKRES mutant proteins frequently maintain a stable fold while retaining functional properties [2, 22]. To confirm this directly, we produced recombinant samples of AGO2-miRNA complexes harboring selected LESKRES mutations. We tested five individual missense mutations that are associated, based on initial clinical findings, with either severe (p.L192P and p.A367P), moderate (p.T357M and p.F182del), or mild (p.G733R) clinical course. All constructs also carried mutations at known AGO2 phosphorylation sites [23–25] to avoid indirect effects associated with differential phosphorylation [2].

**Figure 1.**
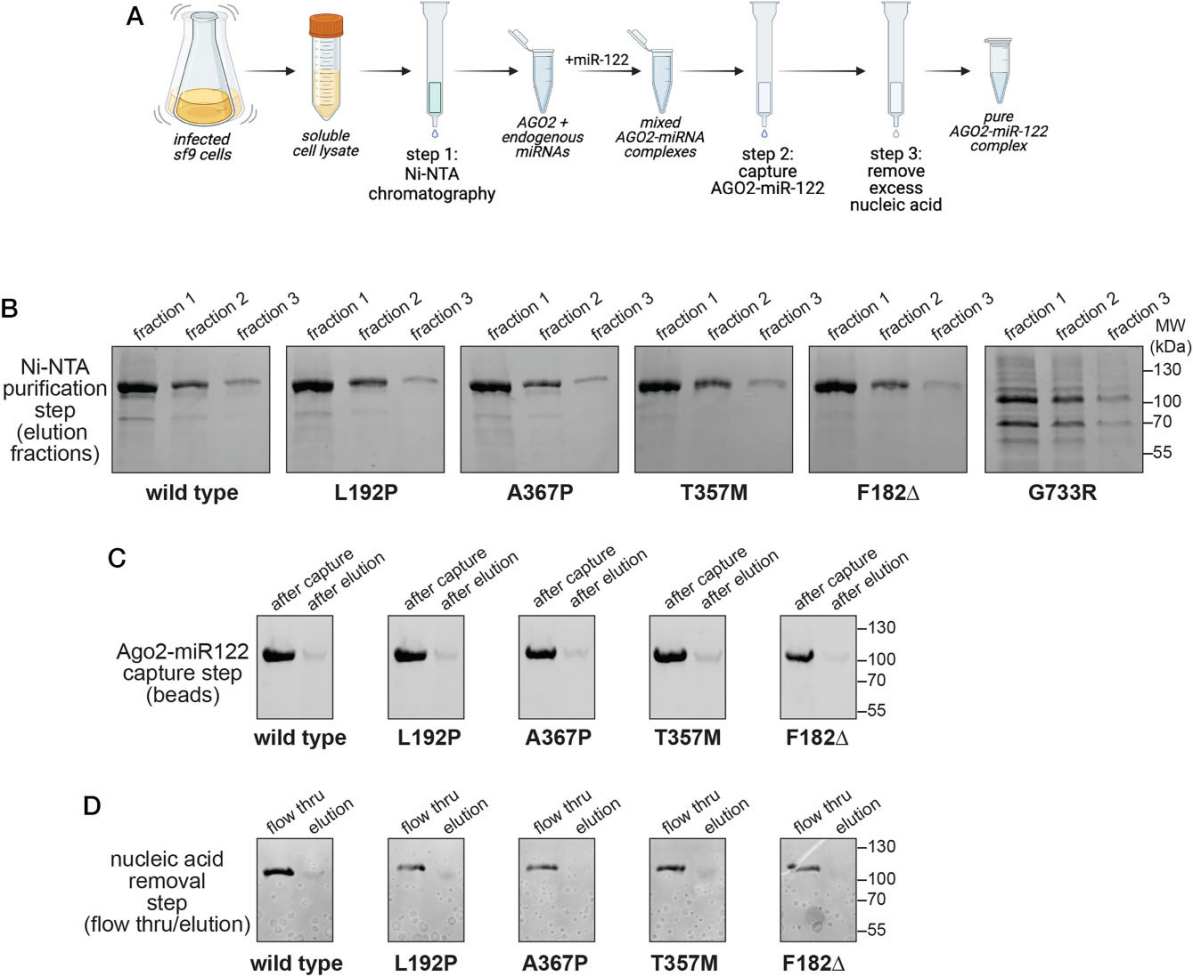
Purification and assembly of mutant AGO2–miRNA complexes. (**A**) Schematic of the purification and assembly process. (**B**) Coomassie-stained, SDS PAGE of eluted fractions collected in succession from Ni-NTA chromatography. (**C**) SDS PAGE of AGO2-miRNA bound to and eluted from an immobilized miR-122-target oligonucleotide. (**D**) SDS PAGE of the final purified samples after flowing through an ion exchange column to remove excess nucleic acids..

His_6_-tagged AGO2 proteins were expressed in Sf9 cells using a baculovirus system, purified by Ni-NTA chromatography, assembled with a synthetic miRNA guide (miR-122), and purified as stable AGO2–miR-122 complexes (Fig. 1A). miR-122 was chosen because this miRNA is biochemically and structurally well characterized [26–28]. The G733R mutant expressed poorly and was lost in the early steps of the preparation, indicating that it does not adopt a stable fold (Fig. 1B). This is supported by earlier data from HEK293T showing the ectopically expressed G733R mutant displays a complete loss of function, including impaired capacity to bind an endogenous miRNA [2]. In contrast, the other mutant AGO2 proteins expressed at levels comparable to the WT. After incubation with miR-122, these mutants were bound to and eluted from an immobilized miR-122 target oligonucleotide with recoveries similar to the AGO2 wild-type (AGO2-WT, Fig. 1B–D). This step requires sequence-specific basepairing between the loaded miRNA and immobilized target oligo [13]. Northern blot of the purified AGO2-miR-122 samples showed equal loading for all preparations, with an AGO2 to miR-122 molar ratio of approximately 1:1 (Supplementary Fig. S1). Thus, except for G733R, all analyzed AGO2 mutants (L192P, A367P, T357M, and F182Δ) adopt a stable and functional fold.

### LESKRES mutants moderately affect target RNA binding affinity of AGO2

**Figure 2.**
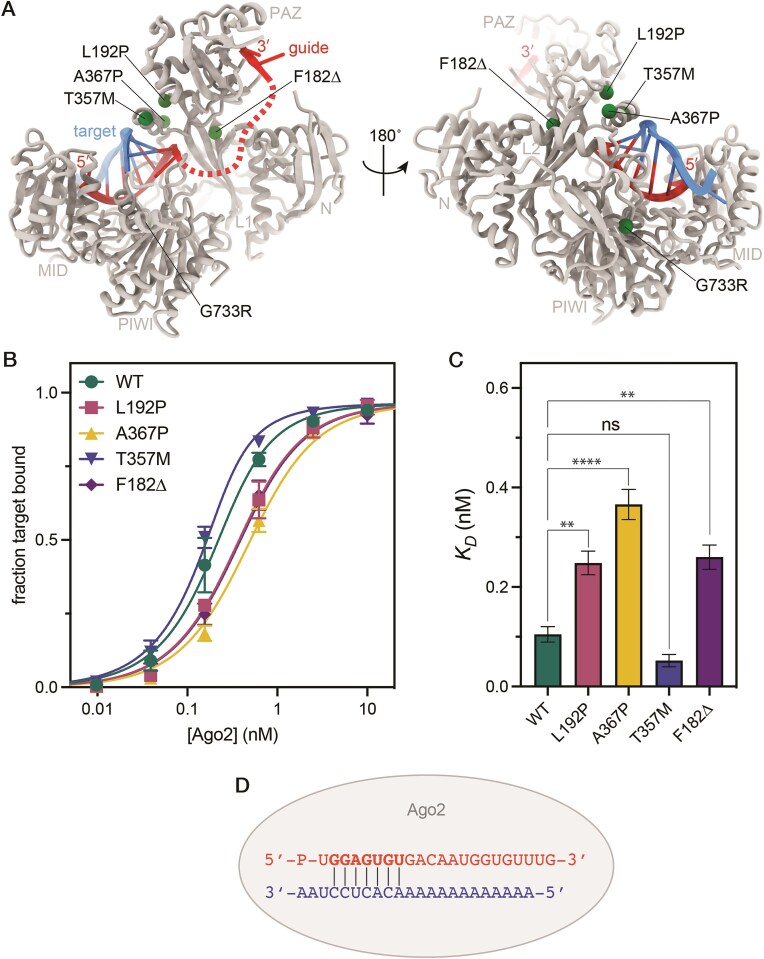
Equilibrium binding of mutant AGO2–miRNA complexes and a target RNA. (**A**) Crystal structure of AGO2 engaging a seed-matched target RNA (PDB 4W5R). Labeled spheres indicate the positions of clinical mutations. L192P, A367P, T357M, and F182Δ cluster within the major hinge involved in RNA binding. G733R is buried inside the core of the PIWI domain. (**B**) Plot of the fraction of target RNA bound as a function of [AGO2-miRNA] for AGO2-WT and the four mutants. Data were normalized to B_max_, the theoretical maximum target bound by each AGO2 sample. (**C**) Plot of the calculated dissociation constant (*K*_D_) values. (**D**) Schematic showing base pairing between the guide RNA (top) and the target RNA (bottom). One-way ANOVA: **** *P* < 0.0001, *** *P*< 0.001, ns ≥ 0.05 (not significant). Error bars indicate SEM.

We next measured the binding affinities of the purified AGO2–miR-122 complexes for a target RNA. We used a short target RNA complementary to the miR-122 extended seed region, which is composed of guide (g) nucleotides g2–g8 counting from the guide RNA 5′ end [7] (Fig. 2A). All mutants effectively bound the target RNA. Plotting the fraction of target RNA bound to AGO2 as a function of AGO2-miR-122 concentration (Fig. 2B) revealed minor differences in the dissociation constant (*K*_D_) values of the AGO2 mutants. Specifically, L192P, A367P, and F182Δ bound the target RNA with affinities 3.8–2.3 times weaker than AGO2-WT (Fig. 2C). The *K*_D_ calculated for T357M was 1.8 times lower than that of the WT, suggesting increased target affinity. However, the difference was not statistically significant (adjusted *P*-value = 0.4988, ordinary one-way ANOVA). We conclude that the selected LESKRES-associated mutations only moderately (∼2–4 fold) alter target binding affinity, but do not abrogate target RNA binding.

### L192P, A367P, and T357M mutants prolong dwell times on target RNAs

**Figure 3.**
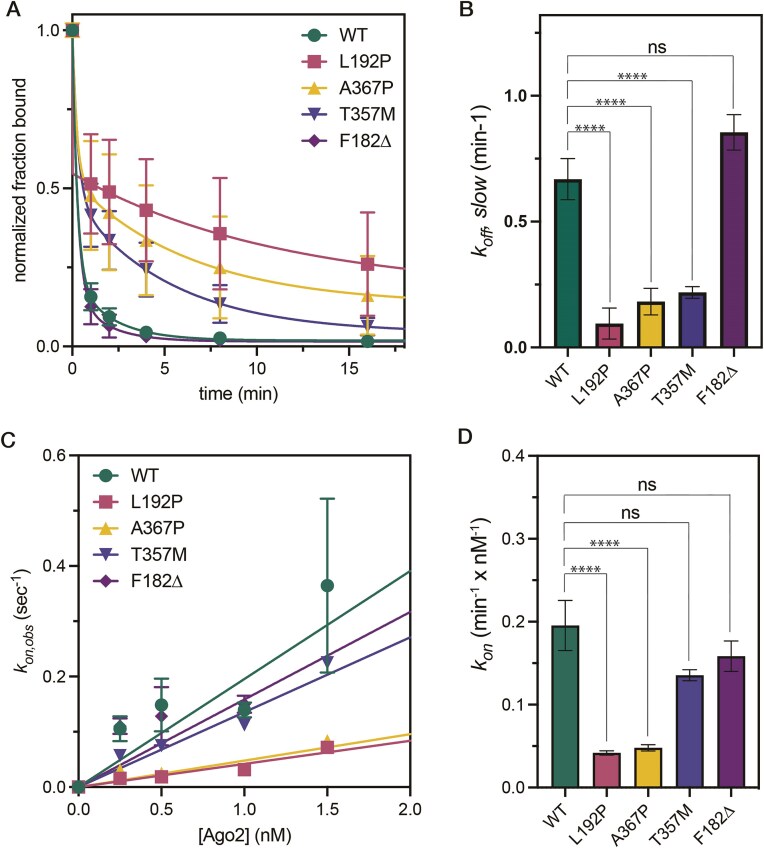
Kinetics of target binding and release by mutant AGO2–miRNA complexes. (**A**) Target release was assessed by measuring the fraction of the target RNA bound to the AGO2–miRNA complex as a function of time. Data points represent the mean of six experimental trials. (**B**) Plot of calculated release rates for the slow step of target release (*k_off, slow_*). (**C**) The observed rate of AGO2-miRNA binding to the target RNA is plotted as a function of [AGO2-miRNA]. Data points represent the average of 3–5 independent trials. (**D**) Comparison of second-order rate constants for binding of AGO2–miRNA complexes to the target RNA.One-way ANOVA: **** *P*< 0.0001, ns *P* ≥ 0.05 (not significant). Error bars indicate SEM.

We next examined how LESKRES-associated missense mutations influence the rates at which AGO2 binds to and dissociates from a target RNA. The first-order rate constant describing target release (*k_off_*) was measured by equilibrating AGO2–miR-122 complexes with ^32^P-labeled target RNA, treating with a vast excess of unlabeled target RNA, and monitoring release of the ^32^P-labeled target RNA from AGO2. A plot of the fraction of ^32^P-labeled target RNA bound (normalized to time zero) versus time shows that the WT AGO2-miRNA-122 complex rapidly released bound target RNAs, as observed previously [15, 28, 29] (Fig. 3A). The F182Δ mutant behaved similarly to AGO2-WT, while the L192P, A367P, and T357M mutants released the target RNA substantially slower.

The target release profiles of the L192P, A367P, and T357M mutants are complex, with a fast phase near the rate of AGO2-WT target release and a slow phase that is ∼20-fold slower (Fig. 3A and B). The amount of target released in the fast-release step varied between experiments from ∼35% to ∼65% of the total bound RNA (Supplementary Fig. S2). We were unable to identify the cause of this variation but did establish that, individually, differences between AGO2 preparations, competitor RNA preparations, buffer preparations, and moderate temperature fluctuations (±5°C) were insufficient to explain the observed variation. Despite the variation between experimental trials, L192P, A367P, and T357M mutants released the target RNA significantly slower than AGO2-WT, with L192P always displaying the slowest release, followed by A367P and T357M. Thus, the L192P, A367P, and T357M mutants display a tendency toward dwelling on bound target RNAs significantly longer than AGO2-WT.

### L192P and A367P mutants reduce target binding rates

We additionally measured the rates of target binding by wild-type and LESKRES mutant AGO2–miR-122 complexes. The ^32^P-labeled target RNA was mixed with various concentrations of each AGO2–miR-122 complex. Bound and free target RNAs were then separated on a filter dot-blot apparatus at various times. The fraction of target RNA bound versus time was plotted to determine an observed binding rate constant (*k_on,obs_*) for different AGO2 concentrations (Supplementary Fig. S2). Observed rate constants were then plotted as a function of AGO2-miR-122 concentration to determine second-order target binding constants (*k_on_*) (Fig. 3C and D).

The L192P and A367P mutants displayed significantly slower target-binding kinetics at all AGO2-miR-122 concentrations tested (Fig. 3C), binding the target RNA at least 5 times more slowly than AGO2-WT. The T357M and F182Δ mutants also appeared to bind at a somewhat slower rate compared to WT, but the measured differences did not reach statistical significance. Notably, all of the observed differences may be underestimated because AGO2-WT target-binding is too rapid to be measured precisely by our method (∼90% of binding was complete after 15 s (Supplementary Fig. S2)). Using published *k_on_* values for AGO2-WT from single-molecule experiments [29, 30], we calculate that the L192P and A367P mutations may reduce target binding rates by as much as 100-fold.

These combined biochemical results indicate that LESKRES-associated mutations affect the kinetics of target binding and release, with the L192P and A367P mutations having the clearest and strongest effects. As both on- and off-rates are strongly reduced, these data are in agreement with the rather moderate changes in target affinity (*K*_D_-values), which may be calculated as the ratio of the two rate constants.

### LESKRES mutations increase mis-targeting *in vitro*

**Figure 4.**
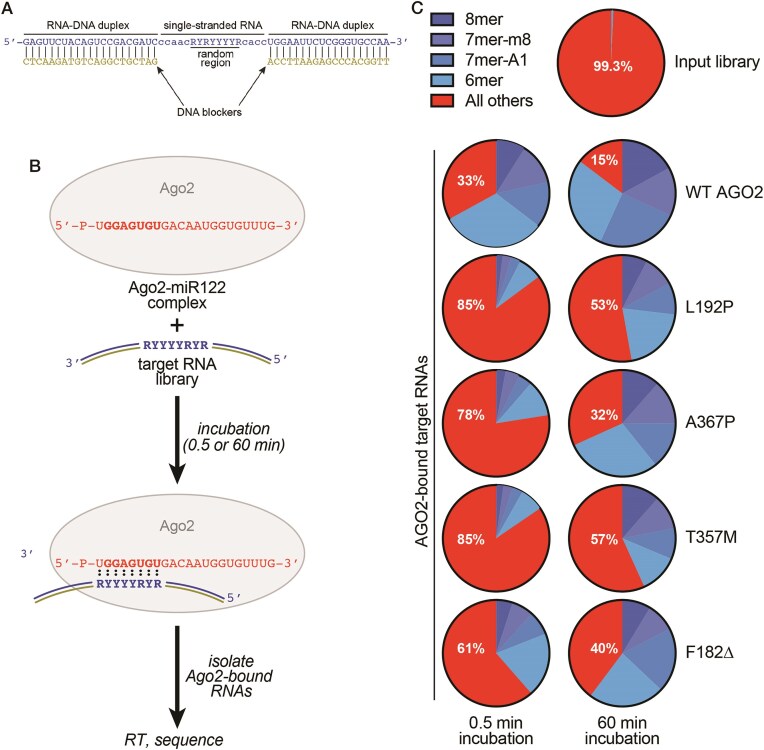
RNA Bind-n-Seq with AGO2 mutants. (**A**) Schematic of the RBNS target used. RNA on top, paired DNA on bottom. R indicates purine; Y indicates pyrimidine. (**B**) Schematic of RNBS experimental workflow. The randomized region in the target RNA library is shown, with Y indicating pyrimidine (C or U) and R indicating purine (A or G). ‘:’ indicates a potential base pair to the miRNA-122 seed region. (**C**) Pie charts showing the fraction of reads containing a seed match (8mer, 7mer-m8, 7mer-A1, or 6mer) and the sum of all reads lacking a seed match in the input library or pulled down by the AGO2-miR complexes after either 30 s or 1 h. Numbers on charts indicate the percentage of reads without a seed match.

Previous work showed that a particular alpha helix within AGO2, termed helix-7, allows AGO2 to avoid stable binding to target mRNAs with mismatches to the miRNA seed region [28]. Because many LESKRES-associated mutations are found in the vicinity of helix-7 or the hinge that enables helix-7 movements, we wondered if these mutations increase mis-targeting. We utilized AGO2 RNA Bind-n-Seq (RBNS) [31] to determine if LESKRES mutations promote mis-targeting. We first synthesized a library of 256 target RNAs, each containing a unique combination of matches or mismatches to the extended seed region of miR-122 (Fig. 4A). The constant regions flanking the variable sequence of the target RNAs were annealed to complementary DNA oligonucleotides to limit both the formation of RNA secondary structure and non-canonical binding interactions [31]. The RNA library was then mixed with WT or mutant AGO2–miR-122 complexes and bound RNAs were isolated by filter-binding after either 30 s or 1 h. AGO2-bound target RNAs were then recovered and identified by RNA-seq.

RNA-seq results showed that the combined total number of RNAs containing one of the four types of canonical seed-matched sites (8mer, 7mer-m8, 7mer-A1, and 6mer) [32] comprised 0.7% of the input target library (Fig. 4B, Supplementary Table S4). Compared to the input library, sequences matching the miR-122 seed region were enriched 100-fold in the RNA samples bound to AGO2-WT (Supplementary Fig. S3). After the 30-s incubation, 67% of the RNA-seq reads associated with AGO2-WT contained a seed match (Fig. 4B). In the sample incubated with AGO2-WT for an hour, seed-matched targets accounted for 85% of the bound RNAs. By contrast, all LESKRES mutant AGO2–miR-122 complexes showed less enrichment of the seed-matched RNAs. For example, only 15% of the RNAs bound to the L192P AGO2–miR-122 complex after 30 s contained a seed match. Consistent with its slow targeting kinetics, the fidelity of L192P increased over time, but even after 1 h only 47% of the target RNA reads contained a seed match, with the remaining 53% representing off-target binding. The other LESKRES mutants (A367P, T357M, and F182Δ) behaved similarly to L192P. Thus, the mutant proteins failed to distinguish seed-matched from non-seed target RNAs to the level accomplished by WT AGO2. We conclude that all four analysed LESKRES-associated mutations reduce the *in vitro* targeting fidelity.

### A murine cortical neuron culture system for studying the L192P AGO2 variant

**Figure 5.**
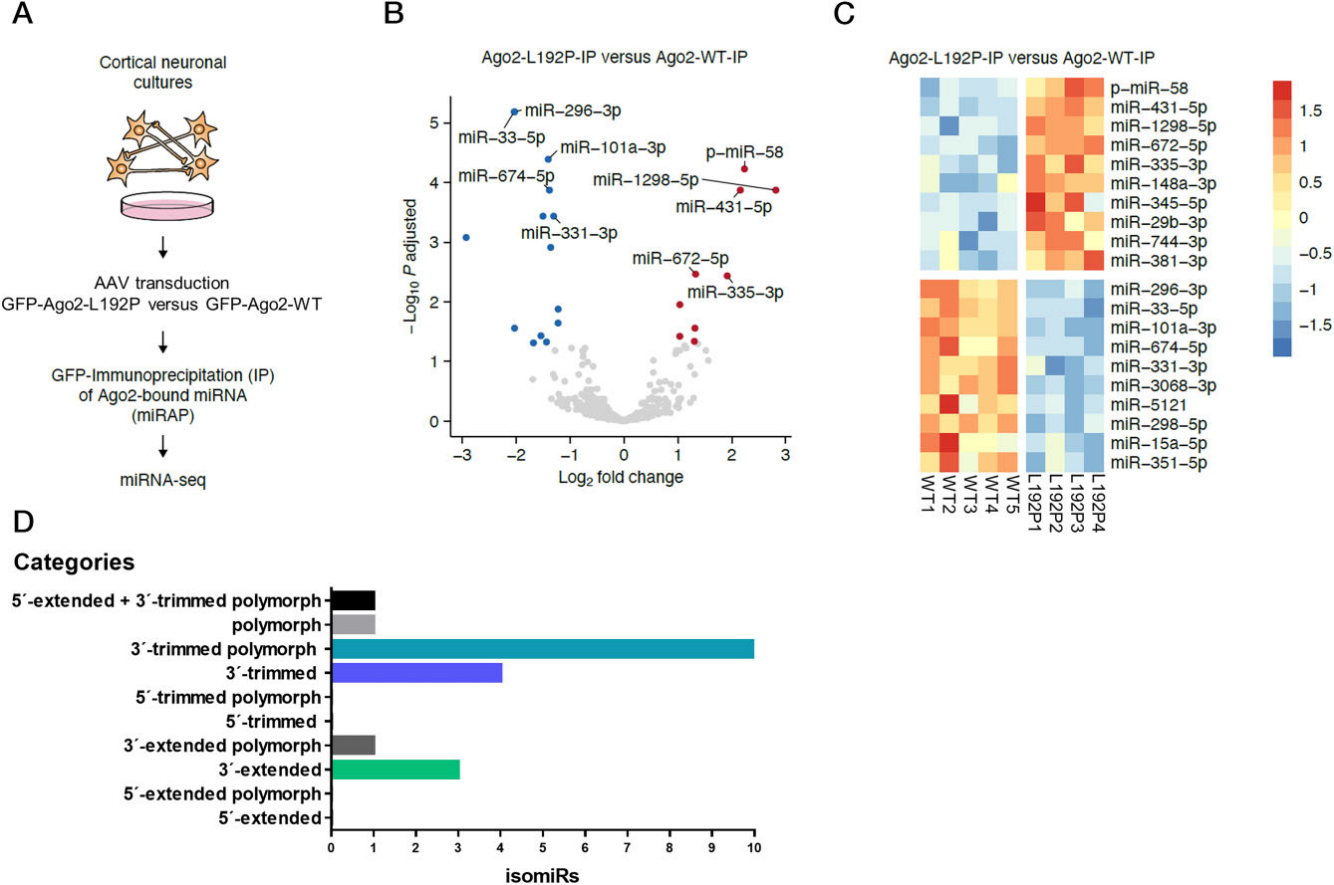
Identification of neuronal miRNAs associated with WT and L192P mutant AGO2 protein. (**A**) Scheme of the experimental approach. Primary cultured murine cortical neurons were transduced with AAVs at 1 DIV to express the GFP-tagged AGO2 variants WT and L192P, or GFP alone. At 12 DIV, GFP-tagged proteins were IP from cell lysates. miRNAs were extracted from IC and IP samples and subjected to RNA-seq analysis. (**B**) Volcano plot analysis of differentially RISC-loaded miRNAs with AGO2-L192P compared to AGO2-WT. Significance was determined by an adjusted *P*-value of *P* ≤ 0.05 and a log_2_ fold change > 1. Decreased miRNAs: blue; enriched miRNAs: red. (**C**) Heatmap of top 10 enriched and top 10 de-enriched miRNAs. (**D**) Several isomiRs were found exclusively in IP samples of the AGO2 L192P variant. Out of the 37 isomiRs that were exclusively bound to AGO2-L192P, the 20 isomiRs with highest average count values were categorized based on the sequence alterations. Around 50% of these isomiRs were 3′-trimmed with nucleotide exchange. The remaining 50% included the other five categories in varying percentages.

The experiments above provide insights into how LESKRES-associated mutations alter the biochemical properties of AGO2 *in vitro*. To gain further knowledge regarding their physiological consequences, we selected the L192P mutant which displayed the most severe functional effects in the analyses described above. As an *in vitro* cellular system, mouse cortical neurons were infected with AAVs to express GFP, GFP-tagged human AGO2-WT, or AGO2-L192P under the control of the neuron-specific hSyn1 promotor [33] (Fig. 5A). Transduction with AAVs induced expression of GFP-AGO2 proteins or GFP in approx. 95% of neurons. Transduced neurons appeared healthy, as judged by staining for the dendritic marker MAP2, with no gross morphological changes or changes in the number of primary dendrites per cell (Supplementary Fig. S4). Staining of transduced neurons for the P-body marker Dcp1a showed that GFP-AGO2-WT and the L192P variant were localized in Dcp1a-positive clusters throughout the soma and along the dendrites (Supplementary Fig. S4). The number of somatic clusters did not differ, whereas the abundance of dendritic clusters was increased upon expression of the L192P variant (Supplementary Fig. S4). Notably, comparable results were observed previously in a different experimental system, i.e. in transfected rat hippocampal neurons expressing L192P, F182Δ, and M364T mutants, but not the loss of function, G733R variant [2]. Thus, increased formation of dendritic P-bodies appears to be a distinctive hallmark of AGO2 mutants with substitutions in the vicinity of helix-7 or the hinge that enables helix-7 movements.

### The L192P mutant alters the composition of RISC-bound miRNAs

We next determined how the L192P mutant impacts the association of AGO2 with cellular miRNAs. We immunoprecipitated (IP) GFP–AGO2–miRNA complexes from AAV-transduced neurons using an anti-GFP antibody. WT and L192P GFP-tagged AGO2 accumulated to similar levels as determined by Western blot of GFP-IP material (Supplementary Fig. S5). Isolation of miRNAs from cell lysates (Input control, IC) and precipitates showed that comparable amounts were coprecipitated from AGO2-WT and L192P-expressing neurons. IP samples of the GFP control neurons showed the lowest miRNA concentration, confirming the specificity of the miRNA tagging and affinity-purification (miRAP [17]) (Supplementary Fig. S5). The specificity of the method for miRNAs derived from the neuronal cell population was further verified by qPCR. For this, the abundance of neuron-enriched miRNA-124 in IC and IP samples was compared to the glia-specific miRNA-21 [34–38]. miR124 was enriched in IP compared to IC samples when GFP-tagged AGO2 variants were expressed. The relative enrichment was determined to be the highest for AGO2-WT (Supplementary Fig. S5).

miRNAs in IC and IP samples from GFP-AGO2 expressing neurons were then identified by NGS. A clustering dendrogram was created to assess the similarity between data from IC and IP samples (Supplementary Fig. S6). Except for WT-IC2, the miRNA datasets hierarchically clustered together depending on sample type (IC versus IP) and AGO2 variant (WT versus L192P), suggesting that miRNAs associated with the L192P-RISC differed compared to the miRNAs present in the canonical WT-RISC.

Differential expression analysis identified alterations in RISC-associated miRNAs associated with the AGO2 variant L192P (Fig. 5A and B). 23 miRNAs were detected to be significantly differentially associated with AGO2-L192P, of which 14 miRNAs were depleted and nine miRNAs were enriched in the mutant AGO2 sample compared to the WT.

### The L192P mutant alters the 3p/5p ratio for some miRNAs

Upon RISC formation, AGO2 receives a miRNA duplex consisting of a 3p and 5p strand, one of which (the “guide” or miR strand) remains anchored at the AGO2 protein, whereas the other (termed passenger or miR*) strand is released and degraded [39–43]. Either the 3p or the 5p strand may be preferentially incorporated [44, 45]. Calculation of 3p/5p strand ratios identified 33 miRNAs with a significant difference (*P* ≤ 0.05) between AGO2-WT-IP and AGO2-L192P-IP. 11 of these miRNAs displayed alterations in 3p/5p ratios specifically in the IP conditions, without displaying altered overall miRNA levels in the input controls (Fig 5). Thus, AGO2-L192P exhibited altered strand selectivity in comparison to Ago2-WT.

### The L192P variant promotes remodeling of the 3′ end of some miRNAs

Further variability in the miRNA complement of cells is achieved by the generation of isomiRs; these are miRNAs that differ from the reference sequence of the corresponding canonical miRNA [46–48]. IsomiRs are miRNAs that have been trimmed or extended at the 5′ or 3′ end, display variations in sequence (polymorph), or a combination of varying length and sequence (mixed isomiRs) [49, 50]. Notably, isomiRs can bind to Argonautes and change the identity of AGO-regulated target mRNAs, making them relevant for the specificity of RISC-mediated gene silencing [51–53]. We analyzed the RNA-seq data regarding the abundance of isomiRs using the miRMaster tool [54]. We identified 37 isomiRs that were bound exclusively to the L192P variant. In contrast, we did not detect isomiRs that were bound exclusively to AGO2-WT. Different modifications were identified, with the majority affecting the 3′ end of canonical miRNAs. Notably, none of the isomiRs analyzed were 5′-edited isomiRs without additional variations at the 3′-end (Fig. 5D and Supplementary Table S3). While we do not know the mechanism(s) leading to the production of these isomiRs, the exclusive presence of 3′ isomiRs indicates that some miRNAs associated with the L192P variant are subject to increased levels of 3′ trimming and tailing, which occurs when the miRNA 3′ end is exposed to cellular exonuclease and polymerase activities [55]. We speculate that the L192P mutant-bound 3′ isomiRs originate from compromised binding at the miRNA 3′ end, making the 3′ end available for tailing and trimming [56]. In summary, the L192P mutant influenced overall miRNA binding, altered strand selectivity, and increased the association of AGO2 with isomiRs in cortical neurons.

### Generation of mice carrying the L192P and G733R variants

As the next step, we decided to further analyze two AGO2 variants *in vivo*, which exhibit opposite extremes in disease severity: L192P and G733R. Both mice were generated using CRISPR/Cas methodology. Founder mice were crossed to C57/Bl6 wild-type mice. Heterozygous mice were generated by breeding heterozygous animals with WT animals, to match LESKRES patients, who also harbor heterozygous *AGO2* variants [2]. The expected Mendelian ratio of 50% of heterozygous offspring was, however, only observed for the Ago2-G733R mice (∼40%). On the contrary, heterozygous Ago2-L192P mice accounted for only about 5–10% of the live births. This observation suggests a possible defect in early embryogenesis; however, surviving animals appeared healthy and showed no obvious signs of disease. Notably, the different numbers of heterozygous offspring further suggest that the L192P variant affects Ago2 function more severely than the G733R variant.

**Figure 6.**
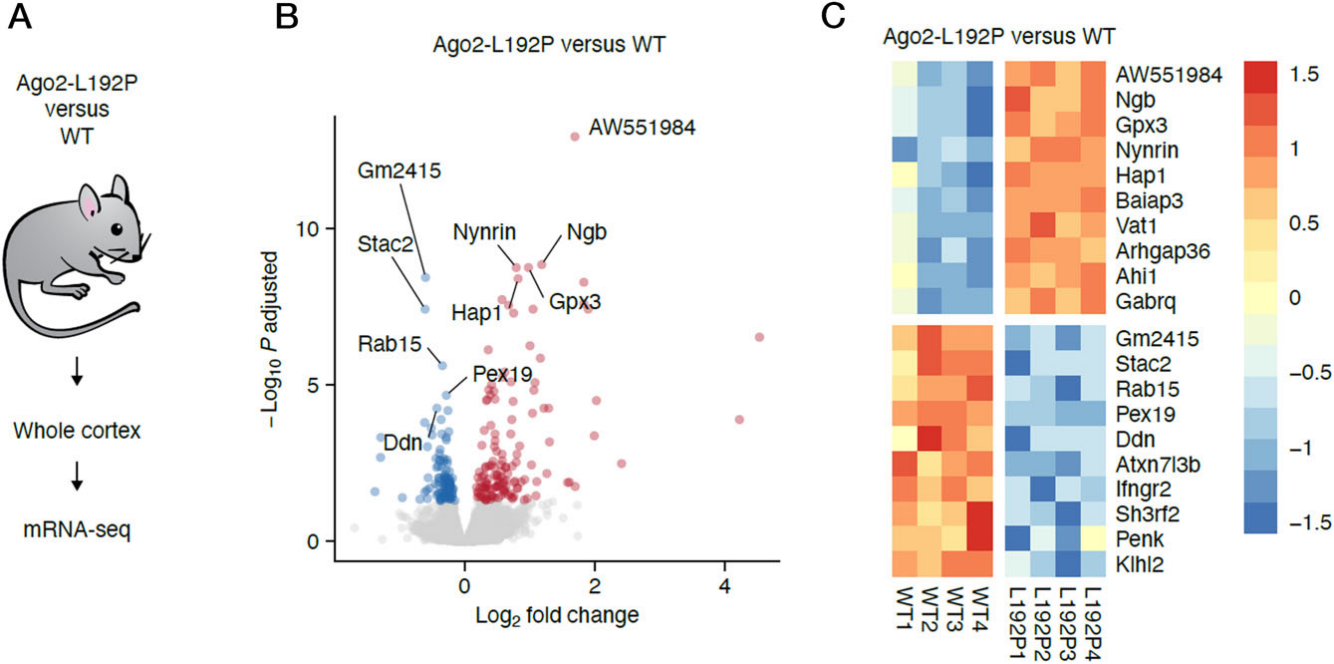
The L192P variant alters the transcriptome of mouse cortex. (**A**) Mice carrying the L192P variant were generated, cortex tissue was analyzed from WT and mutant mice, and subjected to RNA-seq analysis. (**B**) Volcano plot, showing differentially expressed genes. Significance was determined by an adjusted *P*-value of *P* ≤ 0.05, no fold change cut-off was applied. (**C**) Heatmap of the top 10 upregulated and top 10 downregulated mRNAs.

We collected brains from Ago2-L192P, Ago2-G733R, and Ago2-WT mice, isolated total RNA from the cortex of the animals, and subjected this to RNA-seq (Fig. 6). In differential expression analysis of L192P versus WT, we found 247 mRNAs to be significantly regulated with an adjusted *P* value < 0.05. In this analysis, we included all significant candidates regardless of their fold change. Our findings were comparable to previous global transcriptomic alterations observed in primary fibroblasts of patients harboring the p.L192P mutation. While up- and downregulated candidates were detectable in comparable quantities (136 up and 111 down), upregulated mRNAs tended to have higher fold changes (Fig. 6B), potentially indicating a de-inhibition of these transcripts in Ago2-L192P mutant cortices.

In contrast to the L192P data, very few genes were differentially expressed in the Ago2-G733R cortex, leading to a flat volcano plot (Supplementary Fig. S7). This suggests that heterozygous loss-of-function variants have a limited impact on the regulation of gene expression. The few exceptions were several hemoglobin transcripts (*Hba-a1*, *Hba-a2*, *Hbb-bs*), which were significantly decreased in G733R samples. These differences might reflect different amounts of residual blood in the samples. Consistent with this thinking, *Alas2*, an erythroid-lineage cell marker [57], was also reduced in the G733R sample, and loss of Ago2 function was previously shown to be associated with defects in erythroid maturation and hematopoiesis [58, 59]. Alternatively, the observed differences may have arisen from gene expression changes within the cortex as dopaminergic neurons and glial cells can express hemoglobin, which is thought to play a role in protection against oxidative stress and neurodegeneration [60–62].

**Figure 7.**
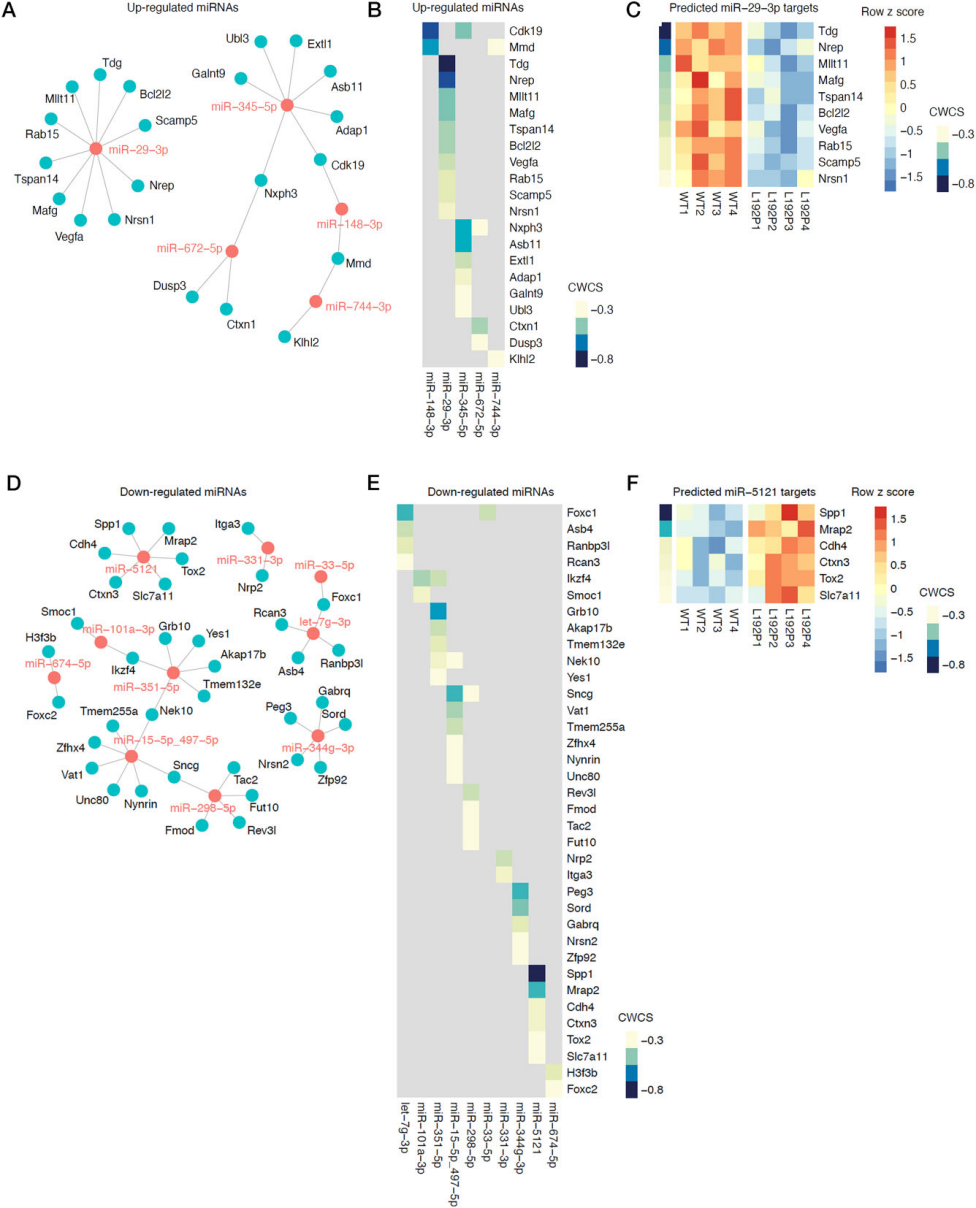
Correlation of altered miRNAs in cortical neurons with altered mRNAs in mouse brain. (**A**) miRNA–mRNA network with miRNAs upregulated in RISC of cortical neurons (red nodes) and their predicted mRNA targets (blue nodes). (**B**) Expression heatmap of the predicted targets of upregulated miRNAs with significant downregulation on mRNA level in the cortex of L192P mutant mice. (**C**) Expression heatmap of predicted targets of miR29-3p. (**D**) miRNA–mRNA network with miRNAs downregulated in RISC of cortical neurons (red nodes) and their predicted mRNA targets (blue nodes). (**E**) Expression heatmap of the predicted targets of downregulated miRNAs with significant up-regulation on mRNA level in the cortex of L192P mutant mice. (**F**) Expression heatmap of predicted targets of miR5121. All predictions are based on TargetScanMouse 8.0. Moderate- and high-confidence targets were defined by a cumulative weighted context score (CWCS) lower than −0.2.

We attempted to integrate changes in AGO2-associated miRNAs upon neuronal transduction with the changes in cortical mRNAs found in the *Ago2* knock-in mice. For miRNAs enriched on AGO2-L192P, we extracted predicted mRNA targets and overlapped these with mRNAs we found downregulated in Ago2-L192P cortices. The resulting network (Fig. 7A) shows target mRNAs that are potentially more strongly inhibited due to increased binding of their regulating miRNAs to AGO2-L192P. Ranking mRNAs by their target prediction strength (Fig 7B), we identified miR-92–3p as a potential regulator of genes, including *Tdg*, *Nrep*, and *Mllt1* (Fig. 7C). Vice versa, for miRNAs de-enriched on AGO2-L192P, we extracted predicted mRNA targets and overlapped these with mRNAs we found upregulated in Ago2-L192P cortices. The resulting network (Fig. 7D) shows target mRNAs that are potentially less strongly inhibited due to decreased binding of their regulating miRNAs to AGO2-L192P. Ranking mRNAs by their target prediction strength (Fig. 7E), we identified miR-5121 as a potential regulator of genes including *Spp1*, *Mrap2*, and *Cdh4* (Fig. 7F). Although being biased by the expected direction of regulation, these analyses provide a framework for explaining cellular and behavioral changes in individuals with AGO2-L192P.

**Figure 8.**
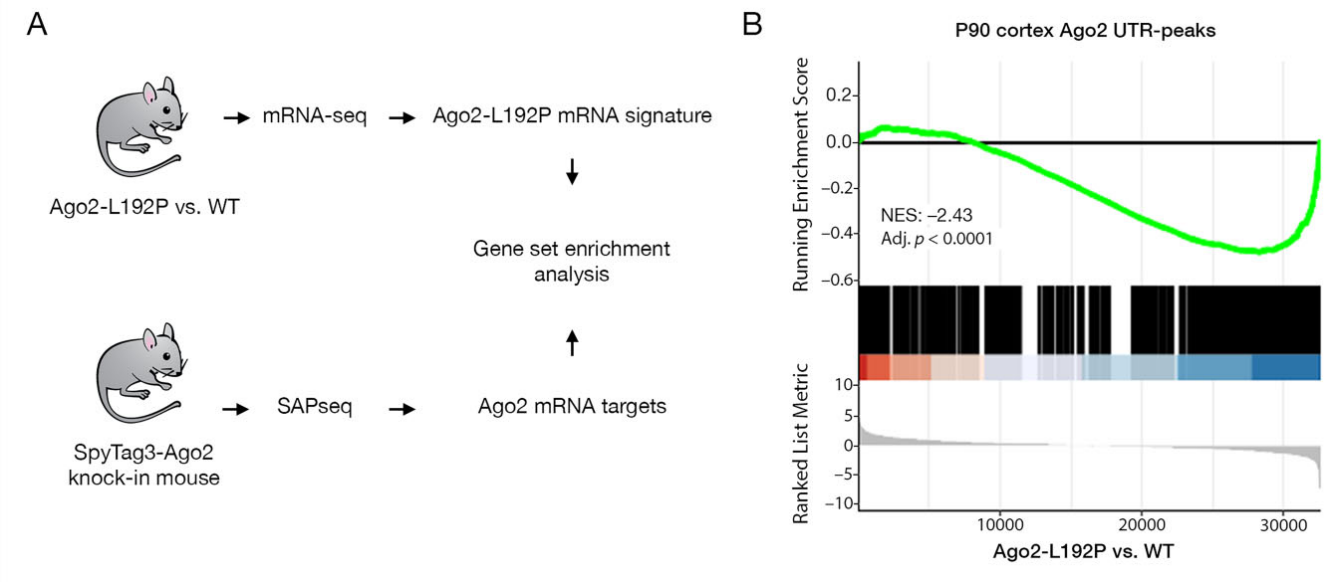
Ago2-associated mRNAs are downregulated in the brain of L192P mutant mice. (**A**) Experimental approach. (**B**) A signature from the mouse cortex RNA-seq dataset was constructed and compared by GSEA with the list of genes that were found to be associated with Ago2 protein in the SAP-seq analysis. In the resulting plot, genes on the left are upregulated in L192P, and genes on the right are downregulated in the L192P mouse cortex.

Finally, we investigated the impact of the Ago2-L192P variant on the regulation of Ago2-associated mRNAs. To do this, we performed cross-linking immunoprecipitation followed by high-throughput sequencing (CLIP-seq) and SAP-seq to map Ago2-bound mRNAs across distinct mouse brain regions [63]. Our analysis focused on transcripts with Ago2 binding sites within their 3′ untranslated regions (3′UTRs) in the mouse cortex. We then applied GSEA to compare RNA-seq data from the cortex of wild-type and L192P mice with the SAP-seq-derived miRNA target dataset. This analysis revealed a strong correlation: mRNAs most strongly associated with Ago2 were selectively downregulated in the cortex of Ago2-L192P mice (Fig. 8). These findings indicate that the L192P mutation leads to over-repression of miRNA-targeted mRNAs in the mouse cortex.

## Discussion

In this study, we performed *in-depth* functional characterization of multiple LESKRES-associated, *AGO2* mutations—p.L192P, p.A367P, p.T357M, p.F182del, and p.G733R—which are associated with variable severity of neurodevelopmental abnormalities. Biochemical characterization revealed distinct impacts on the stability, folding, RNA binding kinetics, and targeting fidelity. Among these, the L192P variant, which is linked to severe clinical phenotype [2], exhibited the most pronounced functional defects. Consequently, we conducted in-depth cell culture and *in vivo* analyses of the L192P variant to further delineate its pathogenic mechanisms.

The G733R mutant exhibited poor expression and was lost early during purification, indicating an unstable and improperly folded protein. This is consistent with previous work showing that the G733R variant is nonfunctional when ectopically expressed in HEK293T cells [2]. This variant abolished shRNA-mediated silencing, phosphorylation of the *C*-terminal serine cluster, binding to GW182, localization to P-bodies, AGO2-mediated slicing, binding to endogenous miRNA, and binding to target mRNAs [2]. Notably, the patient with the heterozygous p.G733R variant and the patient with a heterozygous *AGO2* deletion both exhibited the mildest clinical course within the previously reported LESKRES cohort [2]. Similarly, Ago2-G733R heterozygous mice were born near Mendelian frequencies in heterozygous/WT crosses. Collectively, these findings are consistent with the interpretation that the loss of function of a single *AGO2* allele contributes to a milder clinical phenotype. Conversely, for *AGO2* mutations associated with more severe phenotypes [2], we hypothesize that these mutations may result in detrimental gain of function, as we previously ruled out a dominant-negative effect [2]. These findings contrast those of Duan *et al.*, which found that *alg-1* mutations, corresponding to the ones in the *AGO1* gene, result in dominant-negative effects when expressed in *C. elegans* [22].

The L192P, A367P, T357M, and F182Δ mutants expressed and purified similarly to AGO2-WT, suggesting stable and functional folding. Similar to previous findings, the ability of these mutants to bind miRNAs was not altered. In addition, none of the mutants abrogated target RNA binding, with only moderate differences in target affinity. Notably, several mutants (L192P, A367P, and T357M) released RNA targets significantly slower than AGO2-WT, indicating prolonged interaction with target RNAs. In addition, we observed reduced target-binding kinetics for all tested mutants. However, only L192P and A367P reached statistical significance. As target binding of AGO2-WT occurs near the rate of diffusion, making precise measurements challenging, we expect that we have underestimated the true differences. Notably, our biochemical assays used a short, synthetic miR-122 target RNA to avoid confounding effects from target RNA structure or site accessibility and to minimize alternative guide-target pairing. However, this design may not capture all aspects of target recognition *in vivo*. Additionally, AGO2 *in vivo* is subject to a phosphorylation/dephosphorylation cycle that is not represented in our *in vitro* experiments. Despite these limitations, it is of interest to note that the mutant-specific effects observed in our biochemical assays closely correlate with the clinical severity of patients harboring respective mutations, as p.L192P and p.A367P cause a more severe clinical outcome when compared to p.T357M and p.F182del [2].

In a cellular context, we previously observed increased association of known miRNA target mRNAs with IP AGO2 harboring the L192P, A367P, and F182Δ mutations². This effect may be partially explained by the altered dwell times of these variants on target RNAs. Notably, these same AGO2 mutants exhibited reduced phosphorylation of the *C*-terminal serine cluster [2], suggesting resistance to phosphorylation. Phosphorylation of this region by Casein kinase 1α (CSNK1A1) is known to be stimulated by target RNAs that pair with the miRNA supplementary region (guide nucleotides g12–g16) [64], an interaction that promotes central cleft widening beyond seed pairing alone [27]. Thus, in addition to altering target RNA interactions, these missense mutations may induce conformational changes that impair AGO2 phosphorylation. Given that phosphorylation of the *C*-terminal cluster facilitates target release [24, 25, 64], these findings suggest that, *in vivo*, AGO2 mutants may exhibit prolonged dwell times on target mRNAs beyond those directly measured for the L192P and A367P variants.

Prolonged dwelling time on target RNAs may have several detrimental consequences including mis-targeting and off-target effects. Indeed, all four mutants (L192P, A367P, T357M, and F182Δ) demonstrated increased mis-targeting *in vitro*, e.g. binding to RNAs lacking matches to the miRNA seed region more frequently than AGO2-WT. While extended incubation reduced this mis-targeting, none of the mutants achieved the targeting fidelity of AGO2-WT. Moreover, the prolonged dwell time on target RNAs may be the cause for increased L192P sequestration of mRNAs in P-bodies in distal dendrites. Therefore, we propose the increased target dwell time as the main gain-of-function effect.

Interestingly, all studied AGO2 mutants have amino acid substitutions that are located in the vicinity of helix-7 or the hinge region that enables helix-7 movements of AGO2, which are critical for the protein’s ability to open and close its central cleft. This structural flexibility is proposed to be essential for modulating the base-pairing properties of AGO2-bound guide RNA [28]. Thus, while these mutations do not affect the overall stability of AGO2, they may alter its conformational dynamics, impairing the ability of AGO2 to rapidly bind and disengage from target mRNAs.

Our analysis of GFP-AGO2 expressing primary cortical neurons identified a limited number of differentially RISC-bound miRNAs, some of which were enriched and some were depleted in L192P-RISC. Several miRNAs exhibited altered strand specificity, though no general tendency in binding preference to the passenger or guide strand was observed for the mutant L192P. L192 resides in the main hinge for opening and closing the central cleft of the protein. The L192P variant is likely to change the dynamics of opening and closing of this cleft, which has been proposed to be integral to miRNA-loading [65]. Thus, both the initial loading of miRNA duplexes and the ejection of the passenger strand may contribute to altering the 3p/5p ratios of certain miRNAs.

The AGO2-L192P variant led to an increase in the variety of isomiRs, which differ in their sequence from the corresponding canonical miRNA [46–48]. 37 isomiRs bound exclusively to AGO2-L192P, whereas no isomiR was exclusively associated with AGO2-WT. Almost all of the isomiRs bound to AGO2-L192P exhibited altered 3′-termini. 3′ isomiRs can arise during miRNA biogenesis via altered DROSHA or DICER cleavage or after AGO2-loading if the miRNA 3′-end is exposed to enzymatic attacks [66–68]. Since our previous study showed that the interaction of mutants with DICER is not altered, and the molecular dynamic stimulation suggested loss of anchoring of the guide 3′-end at the PAZ domain [2], we hypothesize that the L192P mutant-bound 3′isomiRs originate from altered AGO2 dynamics. In L192P, the miRNA 3′-end is likely released and exposed to enzymatic attack more often than in AGO2-WT. It should be noted that enzymatic 3′-end trimming and tailing are a hallmark of prolonged engagement with some extensively complementary RNAs [55, 56], again pointing to the prolonged dwelling of the L192P variant on target mRNAs as the primary cause.

Interestingly, Scheper *et al.* determined isomiRs as biomarkers for intellectual disability (ID) and Autism Spectrum Disorder (ASD) in Tuberous Sclerosis complex (TSC), a genetic disorder affecting several organs including the CNS [69–73]. The phenotype of TSC patients includes a broad spectrum of NDDs such as epilepsy, malformations of the cortex, ADHD, ID, and ASD [69, 70]. An isomiR derived from miR409-3p was specifically detected as a biomarker for ID. Considering that the core neurodevelopmental spectrum is similar to the LESKRES, it is important to note that four different miR409-3p derived isomiRs are among the 20 most abundant isomiRs bound exclusively to the L192P mutant.

Finally, cortical transcriptome analysis of mice heterozygous for the *Ago2* p.L192P mutation revealed significant deviations from WT mice. Comparison with a set of Ago2-bound mRNAs from mouse cortex showed that many of these Ago2 target mRNAs are expressed at lower levels than in WT mice. This over-repression of target transcripts is consistent with the L192P mutant’s weakened ability to dissociate from target RNAs. In contrast, apart from differential expression of several hemoglobin-associated genes, transcriptome analyses of mice heterozygous for *Ago2* p.G733R did not identify a major difference in gene expression.

Taken together, our comprehensive analyses collectively show that the more severe clinical outcomes are due to *AGO2* gain-of-function mutations, suggesting prolonged dwell times on target, or possibly off-target, mRNAs as the main mechanistic cause. Our findings have further direct consequences for the accurate evaluation of pathogenicity for future novel *AGO2* missense mutations, and in-frame deletions and duplications. As a first line of analysis, we suggest investigating cellular localization. Mutants failing to localize to P-bodies should be regarded as loss-of-function. Variants localizing to P-bodies should be further investigated in terms of mis-targeting and/or aberrant miRNA and isomiR binding. The development of robust analytical methods to assess and classify novel *AGO2* variants will be a major goal of our future studies.

Rapid and precise determination of the pathogenicity, along with the mode of action, of newly identified variants has significant implications for future clinical interventions. It is conceivable that patients harboring loss-of-function mutations might benefit from interventions aiming to increase the expression of the unaltered, WT allele. Contrarily, in patients harboring gain-of-function mutations, interventions targeting the mutant alleles may ameliorate the severity of clinical signs and symptoms, thereby resulting in milder clinical outcomes. Clearly, such intervention should first be investigated for efficacy and safety in physiological environments. Thus, our labs are currently initiating pre-clinical studies in patient-derived iNeurons.

In summary, this study further highlights the distinct biochemical consequences of different *AGO2* mutations and correlates these with their clinical severity. The loss-of-function G733R mutant results in milder phenotypes, while gain-of-function effects in the L192P, A367P, T357M, and F182Δ mutants lead to more severe phenotypes. Our results suggest a model wherein mutations that alter the AGO2 conformational dynamics impair the exquisite ability of AGO2 to shape the binding properties of its miRNA guide [29], leading to the mis-regulation of target mRNAs and a variety of neurodevelopmental consequences on the organismal level.

## Supplementary Material

gkaf1002_Supplemental_Files

## Data Availability

Mouse cortex sequencing data have been deposited in GEO under the following accession numbers: GSE294381 (L192P RNA-seq), GSE294382 (L192P small RNA-seq), GSE308750 (G733R RNA-seq), and GSE308776 (SuperSeries).
