# Supplementary material for: Dysregulation of AGO2-miRNA dynamics underlies the AGO2-associated Lessel–Kreienkamp syndrome
Source: Nucleic Acids Res. 2025 Oct 16;53(19):gkaf1002. doi: 10.1093/nar/gkaf1002 (PMC12529666; doi:10.1093/nar/gkaf1002)
Supplement: gkaf1002_Supplemental_Files [file gkaf1002_supplemental_files.zip › Supplementary_information-v7.docx]

**Supplementary information**

**Table S1**. Sequences and length of selected isomiRs identified as Ago2-L192P-associated. The RNA sequencing data of miRAP IP samples from Ago2-WT and Ago2-L192P were analyzed using the miRMaster tool to identify Ago2-loaded isomiRs. 37 isomiRs were bound exclusively to the L192P variant. The 20 isomiRs with highest count values were classified as different types of isomiRs. Nucleotide exchanges (nuc.ex) are indicated as position: original base -> isomiR base. The sequences and lengths of isomiRs are shown.

| isomiR | Category | Sequence | Length  in nucleotides (nt) |
| --- | --- | --- | --- |
| miR-409-3p 2T | 3´-extended | GAAUGUUGCUCGGUGAACCCCUUU | 24 nt |
| let-7a-5p 2T 22:T->A | 3´-extended nuc.ex | UGAGGUAGUAGGUUGUAUAGAUAU | 24 nt |
| let-7c-5p 3T 24:G->T | 3´-extended nuc.ex | UGAGGUAGUAGGUUGUAUGGUUUAU | 25 nt |
| miR-138-5p 3T 25:G->T | 3´-extended nuc.ex | AGCUGGUGUUGUGAAUCAGGCCGUUU | 26 nt |
| miR-125a-3p -5T | 3´-trimmed | ACAGGUGAGGUUCUUGG | 17 nt |
| miR-1298-3p -3T | 3´-trimmed | CAUCUGGGCAACUGAUUGA | 19 nt |
| miR-1298-5p -2T | 3´-trimmed | UUCAUUCGGCUGUCCAGAUG | 20 nt |
| miR-135a-1-5p -4T | 3´-trimmed | UAUGGCUUUUUAUUCCUAU | 19 nt |
| miR-135a-2-5p -4T | 3´-trimmed | UAUGGCUUUUUAUUCCUAU | 19 nt |
| miR-148b-5p -3T | 3´-trimmed | GAAGUUCUGUUAUACACUCAG | 21 nt |
| miR-335-3p -3T | 3´-trimmed | UUUUUCAUUAUUGCUCCUG | 19 nt |
| miR-411-5p -3T | 3´-trimmed | UAGUAGACCGUAUAGCGU | 18 nt |
| miR-431-5p -4T | 3´-trimmed | UGUCUUGCAGGCCGUCA | 17 nt |
| miR-544-3p -5T | 3´-trimmed | AUUCUGCAUUUUUAGCA | 17 nt |
| miR-33-5p -2T 18:G->A | 3´-trimmed nuc.ex | GUGCAUUGUAGUUGCAUUACA | 21 nt |
| miR-409-3p -1T 20:C->T | 3´-trimmed nuc.ex | GAAUGUUGCUCGGUGAACCCU | 21 nt |
| miR-409-3p -4T 17:C->A | 3´-trimmed nuc.ex | GAAUGUUGCUCGGUGAAC | 18 nt |
| miR-672-5p -2T 20:T->A | 3´-trimmed nuc.ex | UGAGGUUGGUGUACUGUGUGA | 21 nt |
| miR-672-5p 22:A->T | polymorph | UGAGGUUGGUGUACUGUGUGUGU | 23 nt |
| miR-409-3p 1F -4T 17:C->A | 5´-extended + 3´-trimmed nuc.ex | CGAAUGUUGCUCGGUGAAA | 19 nt |

**Table S2. DNA oligonucleotides used in this study**

| Name | Sequence (5'🡪3') |
| --- | --- |
| **Ago2 Mutagenesis Primers** |  |
| L192P-5'frag_fwd | aaaacctgtattttcagggcgccATGTACTCGGGAGCCGGC |
| L192P 5'frag_rev | ggcccccgccAGGAGGGTTAGAGCAGCC |
| L192P 3'frag_fwd | taaccctcctGGCGGGGGCCGAGAAGTG |
| L192P 3'frag_rev | caagcttggtaccgcatgcctcgagTCAAGCAAAGTACATGGTGCGCAGAG |
| A367P-5'frag_fwd | aaaacctgtattttcagggcgccATGTACTCGGGAGCCGGC |
| A367P-5'frag_rev | ctagcagtcgGTCTGATCATGGTTGAGGTCTGATTG |
| A367P-3'frag_fwd | atgatcagacCGACTGCTAGGTCGGCGC |
| A367P-3'frag_rev | caagcttggtaccgcatgcctcgagTCAAGCAAAGTACATGGTGCGC |
| T357M-5'frag_fwd | aaaacctgtattttcagggcgccATGTACTCGGGAGCCGGC |
| T357M-5'frag_rev | ctgattgtccATTAATTTTTTAATACATCTTTGTCCTGCCACAATG |
| T357M-3'frag_fwd | aaaaattaatGGACAATCAGACCTCAAC |
| T357M-3'frag_rev | caagcttggtaccgcatgcctcgagTCAAGCAAAGTACATGGTG |
| F182del-5'frag_fwd | aaaacctgtattttcagggcgccATGTACTCGGGAGCCGGC |
| F182del-5'frag_rev | cggacgcggtGAAGGAGCGGCCCACGGG |
| F182del-3'frag_fwd | ccgctccttcACCGCGTCCGAAGGCTGC |
| F182del-3'frag_rev | caagcttggtaccgcatgcctcgagTCAAGCAAAGTACATGGTGCGCAG |
| Capture oligo | Biotin-TCTCGTCTAACCATGCCAACACTCCAACTCT |
| DNA competitor | Biotin-AGAGTTGGAGTGTTGGCATGGTTAGACGAGA |
| RBNS 5' Blocker | GATCGTCGGACTGTAGAACTC |
| RBNS 3' Blocker | TTGGCACCCGAGAATTCCA |
| RT primer | CCTTGGCACCCGAGAATTCCA |
| **CRISPR DNA oligos** |  |
| HDR template L192P | TTCCTTCTTCACTGCATCTGAAGGCTGTTCCAACCCT**CCA**GGTGGGGGCAGAGAAGTGTGGTTTGGCTTCCATCAGT**CTG**TCCGACCTTCTCTTTGGAAAATGATGCTGAATATTGATGG |
| Ago_L193P_for | TCTGAGTGTAGCTATGCCTGCTATGATGATG |
| Ago_L193P_rev | CAGGCTGGTATCACATCAGAGCTGTGC |
| HDR template G734R | TGCACAGGTAGAAGTCAAACTCAGTGGGGTGGGTGATCTTCGTGTCCACGGTTGTGC**G**TGCGGGAATGTTCCCACTCTTCCCAACCTGCAACAGCACAGAGGGCTCATCAGCTGTGGCAG |
| Ago_G734R_for | actgtgcttgactgtagcatcagg |
| Ago_G734R_rev | tcctgcagtcagtcccacctttta |

**Table S3. RNA oligonucleotides used in this study**

| Name | Sequence (5'🡪3') |
| --- | --- |
| Guide RNA | P-UGGAGUGUGACAAUGGUGUUUG |
| Seed-matched target RNA | AAAAAAAAAAAAACACUCCUAA |
| RandomTarget RNA | GAGUUCUACAGUCCGACGAUCccaacRYRYYYYRcaccUGGAAUUCUCGGGUGCCAA |

“P” indicates 5' phosphate; “R” indicates purine (A or G); “Y” indicates pyrimidine (C or U)

**Table S4. Ago2 RNA bind-n’-seq data.** First tab: read counts for each target sequence in each data set. Numerical values indicate the number of times each target sequence was observed. Second tab: enrichment compared to input. Fold enrichment was calculated by first calculating the reads per million (rpm) value for each sequence within each sample, and then dividing the rpm for each sequence in each sample by the rpm value of the corresponding sequence in the input library.

**Fig. S1. Northern blot of Ago2-miR-122 complexes.** Left half: synthetic miR-122 standards. Right half: each lane contains 1 µg of purified hAgo2-miR-122 complex from either wild-type (WT) or one of four mutant preparations. Comparison to miR-122 standards indicates that each hAgo2-miR-122 sample contains ~75 ng of miR-122, consistent with a ~1:1 stoichiometry between miR-122 and hAgo2.


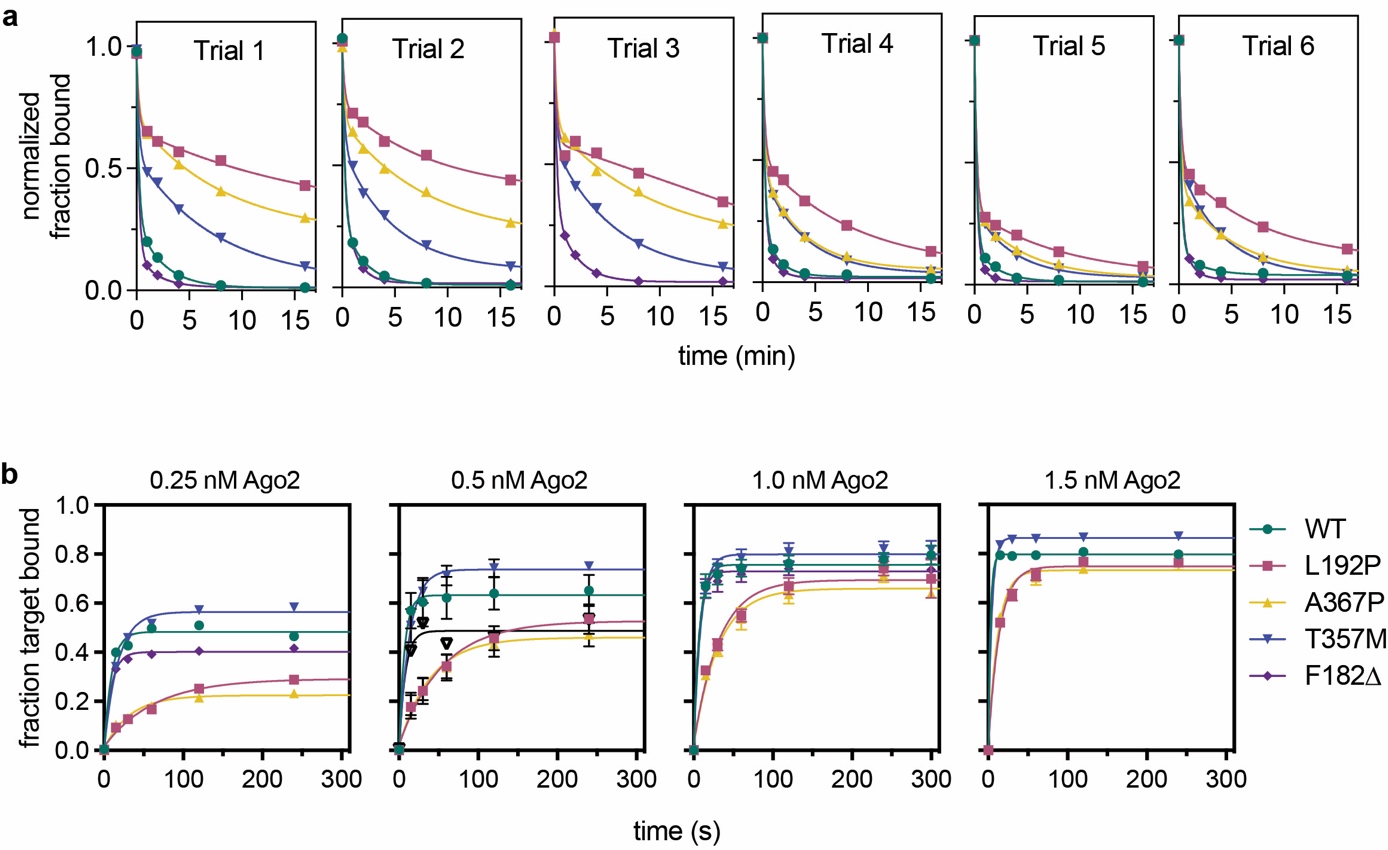


**Fig. S2. Data for independent kinetic trials.** **a.** Target release data. The fraction lost in the fast step varied between trials for undetermined reasons, but the rank order of mutants was the same in all experiments. **b.** Total fraction of target RNAs bound by various concentrations of AGO2-miRNA complexes as a function of time. Data were fit to a single exponential to determine the observed binding rate constant (*k_on, obs_*) for each Ago2-miRNA concentration.


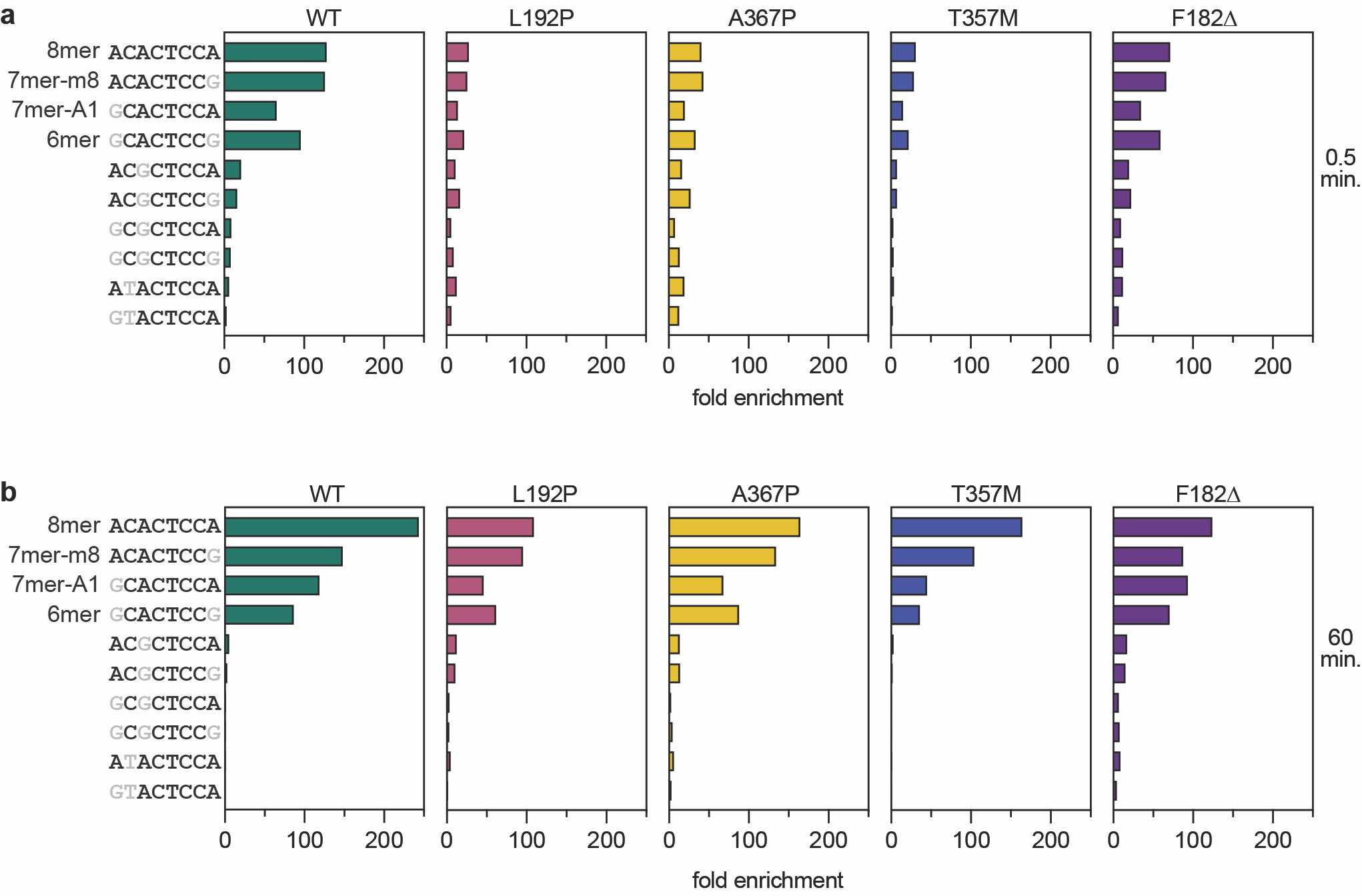


**Fig. S3. RBNS enrichment data.** **a.** The fold enrichment (compared to the input library) for each RBNS data set is shown for the top 10 sequences bound to wild-type Ago2 after 0.5 minutes. Target sequences are shown on the left. Black lettering indicates a match to the seed region, grey lettering indicates a mismatch. Sequences corresponding to the four canonical miRNA target site types (8mer, 7mer-m8, 7mer-A1, and 6mer) are labelled. **b.** Fold enrichment for the same target RNAs as in (a) in the 60-minute incubation data sets.


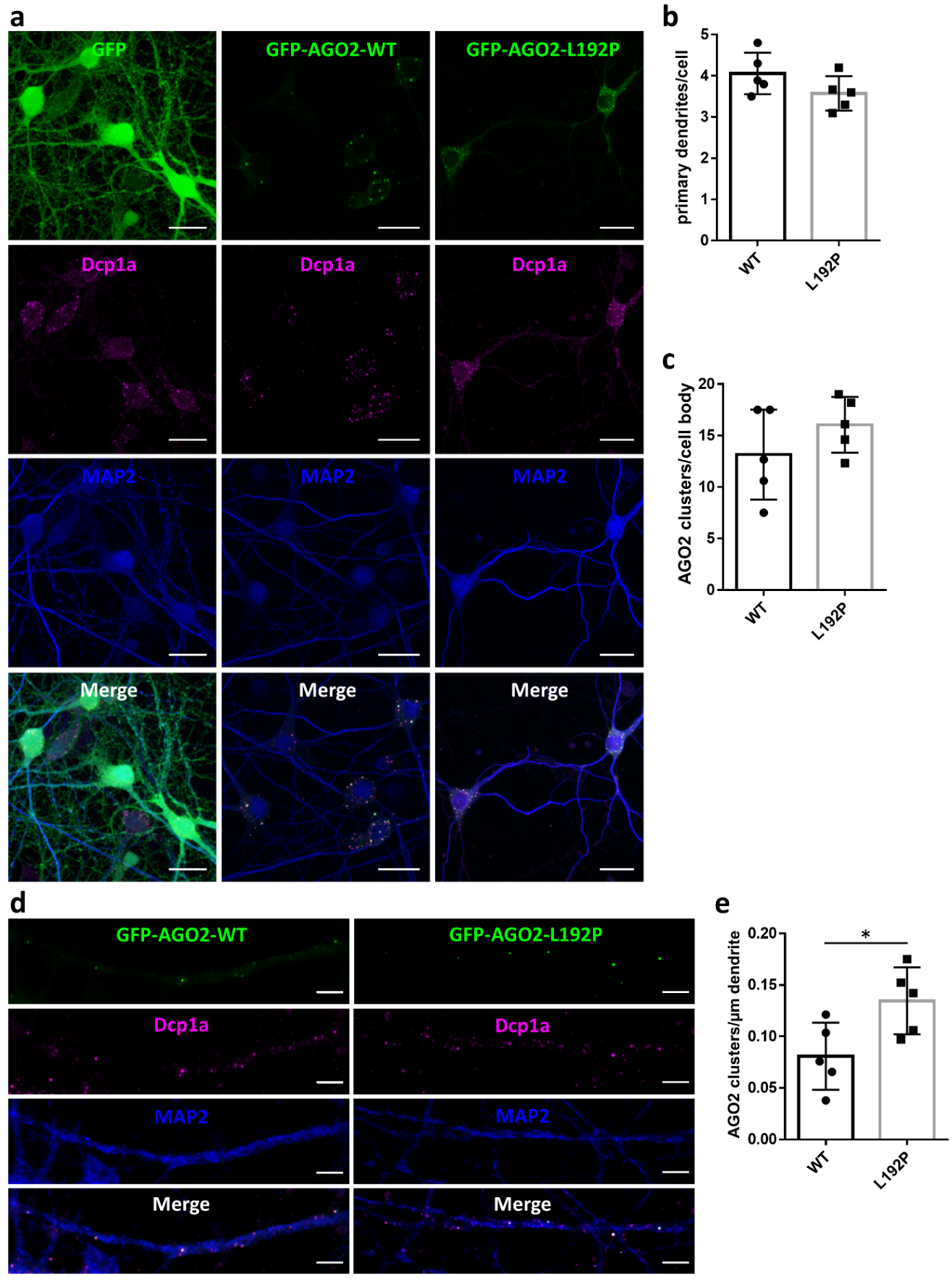


**Fig. S4.** **a.** Confocal microscopy of cortical neurons expressing GFP or GFP-AGO2 variants. In addition to GFP-fluorescence, neurons were stained with antibodies for the P-body marker Dcp1a (magenta) and the dendritic marker MAP2 (blue). Scale bar, 10 µm**.** **b.** Quantitative analysis of the number of MAP2-positive primary dendrites per neuron. **c.** Quantitative analysis of the number of GFP-AGO2 clusters per neuronal cell body**.** **d.** Enlargement of dendritic fragments. Scale bar, 5 µm**.** **e.** quantitative analysis of the number of GFP-AGO2 clusters per dendrite length.


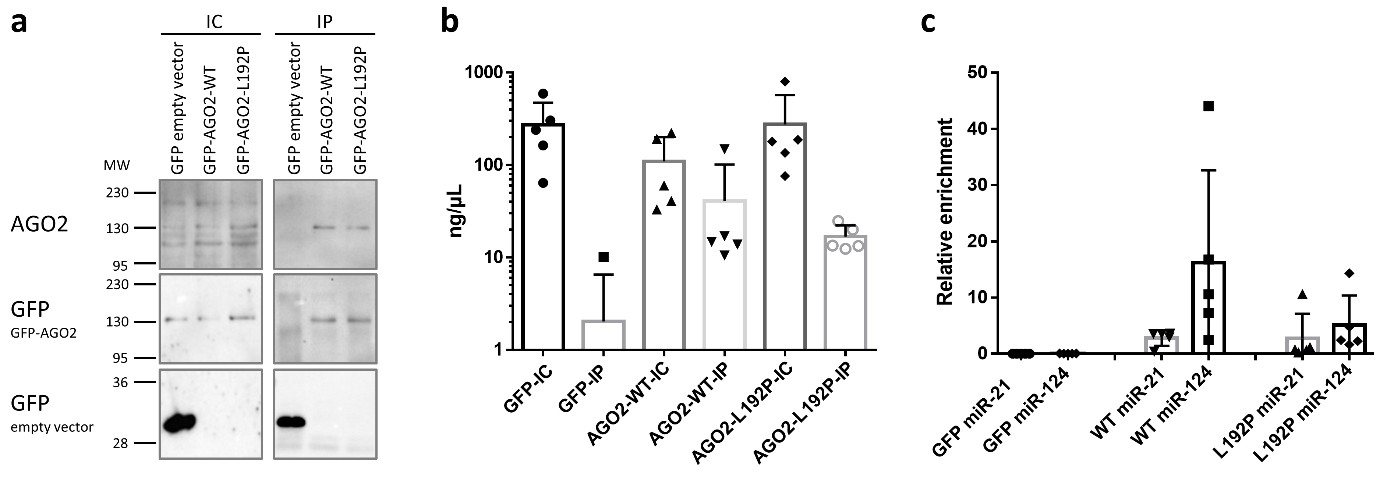


**Fig. S5.** Cortical neurons expressing GFP, or GFP-AGO2 variants, were lysed and subjected to immunoprecipitation (IP) using an anti-GFP antibody. **a.** Western blot analysis of input control (IC) and IP samples, using the antibodies indicated**.** **b.** miRNAs were isolated from IC and IP samples and quantified**.** **c.** qPCR analysis of miRNAs isolated from IP samples, using primers specific for miR-21 and neuronally expressed miR-124. Data are presented as relative enrichment over IC samples.


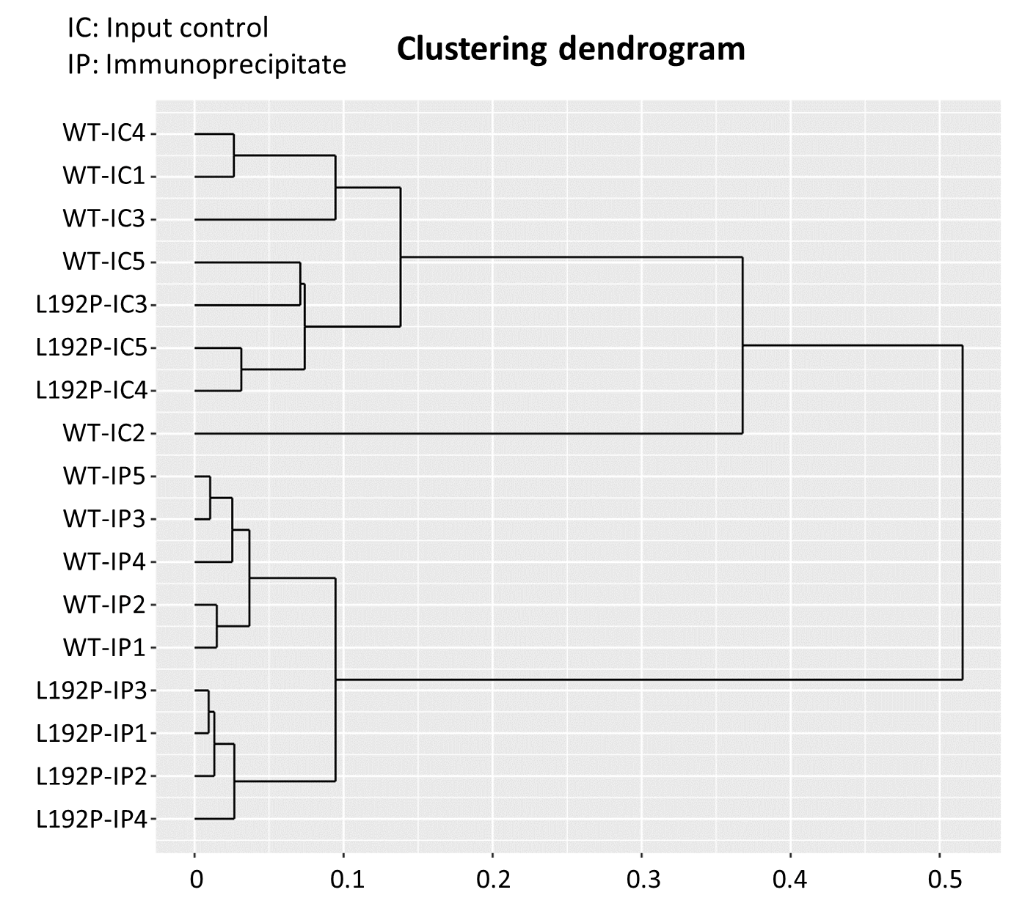


**Fig. S6. Dendrogram depicting clustering of samples in the miRNA sequencing dataset.**

Hierarchical clustering of miRNA profiles from input control (IC) and immunoprecipitated (IP) samples of neurons expressing GFP-tagged AGO2 (WT or L192P). Each branch represents an independent replicate. Except for one outlier (WT-IC2), samples clustered according to sample type (IC vs. IP) and AGO2 variant (WT vs. L192P), indicating that the miRNA composition of L192P-RISC differs from that of WT-RISC.


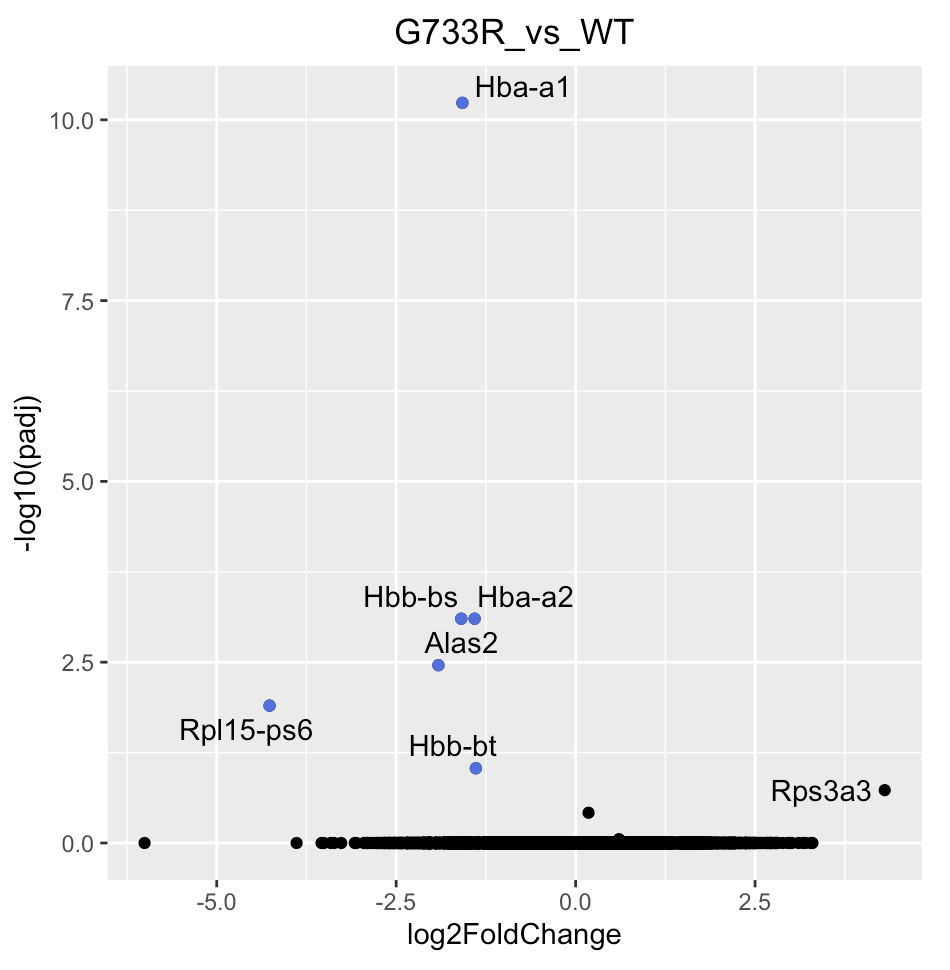


**Fig. S7. Differential gene expression in Ago2-G733R mutant cortex.**
Volcano plot showing results of mRNA-seq comparing cortex samples from heterozygous Ago2-G733R and wild-type (WT) mice. Each dot represents a gene; blue dots denote genes with adjusted *p* < 0.05. Only a small number of transcripts were differentially expressed in G733R cortex, with hemoglobin-related genes (*Hba-a1, Hba-a2, Hbb-bs, Hbb-bt*) and the erythroid-specific gene *Alas2* among the most significantly downregulated.
